# Verminoside from *Pseudolysimachion rotundum* var. *subintegrum* sensitizes cisplatin-resistant cancer cells and suppresses metastatic growth of human breast cancer

**DOI:** 10.1038/s41598-020-77401-7

**Published:** 2020-11-23

**Authors:** Changhu Lee, Hyung Won Ryu, Sahee Kim, Min Kim, Sei-Ryang Oh, Kyung-Seop Ahn, Jiyoung Park

**Affiliations:** 1 Department of Biological Sciences, Ulsan National Institute of Science and Technology (UNIST), Building #110, Rm 501-7, Ulsan, 44919 Republic of Korea; 2 Natural Medicine Research Center, Korea Research Institute of Bioscience and Biotechnology (KRIBB), Cheong-ju si, Chungcheongbuk-do 28116 Republic of Korea

**Keywords:** Cancer, Molecular medicine

## Abstract

Breast cancer is one of the most common cancers in women and is associated with a high mortality rate. The majority of deaths resulting from breast cancer are attributable to metastatic growth; in addition, chemoresistance is a major concern in the treatment of patients with breast cancer. However, limited drugs are available for the treatment of metastatic breast cancer. In this study, the chemoadjuvant effects of a methanolic extract from the leaves of *Pseudolysimachion rotundum* var. *subintegrum* (NC13) and an active component isolated from the plant, verminoside (Vms), were evaluated. Furthermore, their potent anti-metastatic activities were validated in vitro and in vivo in animal models. The anti-metastatic and chemosensitizing activities of NC13 and Vms on cisplatin treatment were found to be partly mediated by suppression of the epithelial–mesenchymal transition of cancer cells. Collectively, our results implied that NC13 and its bioactive component Vms could be developed as effective chemoadjuvants in combination with conventional therapeutics.

## Introduction

Globally, breast cancer is one of the most common cancers and a leading cause of cancer deaths, especially in westernized countries^1^. In 2018, approximately 2.1 million women were diagnosed with breast cancer worldwide, accounting for 25% of cases of cancer in women^2^. Despite the current improvement in diagnosis and therapy, breast cancer remains a major global health burden.

There are a number of chemotherapeutic drugs approved for breast cancer, including cisplatin, docetaxel, doxorubicin, gemcitabine, paclitaxel, and 5-fluorouracil^3^. Various factors affect the efficacy of chemotherapy, including the delivery and penetration of drugs, target cell selectivity, and drug resistance of the tumors^4^. Cisplatin is a platinum-based drug used to treat breast cancer and a wide range of other cancers, including lung, ovarian, brain, and bladder cancers. It is a potent DNA chelator, forming DNA-platinum adducts that subsequently induce apoptosis in cancer cells. Although the initial response to cisplatin is outstanding, resistance to the drug eventually occurs in most patients. When drug resistance occurs during therapeutic treatment, although drug-sensitive cancer cells are initially eliminated, as selected subpopulations become no longer responsive to prolonged treatment, the cancer may reoccur^5^. Several researchers have attempted to overcome cisplatin drug resistance through combination therapies^6^. For example, synergistic effects have been demonstrated with cisplatin combination therapy with other drugs, such as paclitaxel, 5-fluorouracil, doxorubicin, and gemcitabine^7,8^. Besides chemoresistance, drug toxicity is a major problem in current cancer therapy. Most patients with cancer who are administered chemotherapeutic agents experience harmful side effects^9^. For example, adverse effects due to cisplatin treatment include neurotoxicity, nephrotoxicity, and cardiotoxicity^10^. These side effects may make it difficult for patients to continue chemotherapy and can compromise their quality of life^11^. Therefore, the development of new adjuvants used for combination therapy is crucial to improve the efficacy and safety of conventional chemotherapy.

Over the past few decades, natural compounds have been shown to exert chemotherapeutic and chemopreventive effects in several cancers; some of these natural compound-derived chemotherapeutic drugs have been approved for treatment, such as paclitaxel (Taxol), first extracted from the bark of the western yew tree (*Taxus brevifolia*)^12^, and homoharringtonine, originally extracted from *Cephalotaxus harringtonia*^13^. Furthermore, a number of natural compound-based chemotherapeutic drugs are currently in clinical trials; examples include plinabulin, plitidepsin (phase III clinical trials), AGS-16C3F, polatuzumab vedotin, and PM184 (phase II clinical trials)^14^. In addition, some natural compounds are considered to be potential candidate adjuvants for combination chemotherapy. For example, the combination of cisplatin with wogonin or triptolide was shown to have synergetic effects in treating several cancers^15^. Verminoside (Vms) is isolated from a methanolic extract of the leaves of *Pseudolysimachion rotundum* var. *subintegrum* (NC13), which is a known Asian traditional medicine used for the treatment of inflammatory diseases such as bronchitis and asthma^16^. Recent studies have shown that NC13 exerts anti-inflammatory effects in various diseases, especially in chronic obstructive pulmonary disease^17^. In this study, we evaluated the efficacy of NC13 and Vms as chemoadjuvants for cisplatin and further validated the anti-cancer effects of these combination therapeutics in vitro and in vivo in mouse models of breast cancer.

## Methods

### Plant material and isolation of bioactive components

The plant samples were obtained from the Korea Research Institute of Bioscience and Biotechnology (KRIBB). The voucher specimen (KRIB 0020697) was deposited in the KRIBB, Cheong-ju si, Chungcheongbuk-do, Korea. The isolation of Vms from NC13 was described previously^18^. In summary, the dried stems and leaves of NC13 (2.0 kg) were extracted three times with methanol (MeOH) at room temperature to obtain 198.7 g of solid extract. The MeOH extract (1.0 g) was subjected to preparative reverse phase chromatography (GRACE C18, 10 µm, Grace Davison Discovery Sciences, Hesperia, CA, USA) and was eluted isocratically using 25% MeOH in a H_2_O solution. The fractions (Frs. 1–6) were collected and concentrated in a rotary evaporator under reduced pressure. The Fr. 4 was chromatographed using a middle performance liquid chromatography column with RPC-18 (Zeoprep C18, 10 μm, 20 × 250 mm, Zeochem, Louisville, USA) and eluted using a gradient mixture of CH_3_OH-H_2_O (20% → 100%) to yield five subfractions (F4a–4e). The fraction F4b was separated by semipreparative high-performance liquid chromatography (HPLC) (Atlantis T3, 5 μm, 19 × 250 mm, Waters, Milford, USA, 18% CH_3_CN in H_2_O) to afford isovanillyl catalpol (NC106, 10.7 mg). The F4c was separated by semipreparative HPLC (Synergy Polar-RP 4 μm, 21.2 × 250 mm, Phenomenex, Torrance, CA, USA, 22% CH_3_CN in H_2_O) to afford Vms (23.5 mg) and picroside II (NC105, 12.4 mg). The F4d was separated by Sephadex LH-20 (Pharmacia Biotech AB, Uppsala, Sweden, 90% CH_3_OH in H_2_O) to afford 6-*O*-veratroyl catalpol (NC107, 6.1 mg).

### Materials

Cisplatin (P4394) was purchased from Sigma (USA). BCA protein assay kit (23225) was purchased from Thermo-Scientific (USA). Nitrocellulose membranes (1060003) were purchased from GE (USA). E-cadherin (#3195), Snail (#3879), Slug (#9585), Vimentin (#5741), p-ERK (#9106), ERK (#4695), and pan-keratin (#4545) antibodies were purchased from Cell signaling technology (USA). β-Actin (Sc-47778) antibody was purchased from Santa Cruz (USA). Ki-67 antibody (ab15580) was purchased from Abcam (USA). IR800 dye-conjugated rabbit (#926-32213) and mouse (#926-32212) secondary antibodies were purchased from Li-COR Biosciences (USA). Secondary antibody labeled with Cy3 (#711-165-152) was purchased from Jackson ImmunoResearch (USA) and Biotin-rabbit IgG (656140) from Invitrogen, and streptavidin-HRP (P0397) from DAKO (USA).

### Cell culture

MCF7 and MDA-MB-231 breast cancer cells were acquired from the American Type Culture Collection (ATCC, VA, USA). MCF7 and MDA-MB-231 cells were cultured in DMEM supplemented with 1% penicillin/streptomycin and 10% fetal bovine serum, all of which were obtained from Hyclone (MA, USA), and maintained in a 5% CO_2_ incubator at 37 °C.

### Cell viability assay

Cell viability was measured by performing MTT (3-(4,5-dimethylthiazolyl-2)-2,5-diphenyltetrazolium bromide) assays (Invitrogen, CA, USA). Cells (8 × 10^3^ cells/well) were seeded in 96-well plate. On day after, either NC13 or Vms were treated for 44 h, and then the MTT reagent was added for another 4 h. After removing culture media, formazan was dissolved in 100 μL of DMSO, and absorbance was measured at 590 nm using a microplate reader (Infinite M1000, Tecan).

### Wound healing assay

MDA-MB-231 breast cancer cells were seeded in 96-well ImageLock plates (Essen BioScience, MI, USA) and incubated at 95–100% confluency. Wound-Maker (Essen BioScience) was used to make a scratch in each well, and the cells were washed two times. After cells were treated with compounds such as cisplatin, Rosi, NC13 or Vms in serum free media, cellular migration images were taken every 2 h by using the IncuCyte Live-Cell Imaging System (Essen BioScience).

### Western blot analysis

Cell lysates were prepared with NETN buffer (1% NP-40, 1 mM EDTA, 20 mM Tris–Cl, pH 7.5, 100 mM NaCl, 5 mM sodium pyrophosphate, 1 mM sodium orthovanadate, and 50 mM NaF), and protein concentration was measured using the BCA protein assay kit. Approximately 40 μg of proteins were resolved using 10% sodium dodecyl sulfate–polyacrylamide gel electrophoresis and transferred to nylon membranes. After 1 h blocking in 5% skim milk, the membranes were further incubated with the primary antibody overnight at 4 °C. The membranes were then washed three times with TBST buffer and incubated in IR800 dye-conjugated secondary antibodies for another hour. Immunodetection was performed using an Odyssey CLx scanner (Li-COR Biosciences).

### Immunofluorescence

MDA-MB-231 and MCF7 cells were seeded on coverslips overnight. After the indicated treatment, cells were washed with phosphate buffered saline (PBS) and fixed with 4% paraformaldehyde in PBS for 30 min. After rinsing, fixed cells were blocked with NH_4_Cl for 5 min and permeabilized with 0.1% Triton X-100 in PBS. Coverslip samples were incubated with the primary antibody and Vimentin overnight at 4 °C, followed by the secondary antibody labeled with Cy3 for 1 h at room temperature. DAPI was co-stained. Images were acquired with an Olympus Fv-1000 confocal microscope.

### Dosage information

For in vitro experiments, we tested several doses of NC13 or Vms, and thus, 5 μg/mL and 10 μM (5.244 μg/mL) were selected as the optimal concentrations for NC13 and Vms based on *CompuSyn* analysis, respectively (also see Supplementary Fig. S5). The in vivo dosage of NC13 and Vms was tested at 20 mg/kg based on our previous study^17^ of their anti-inflammatory effects. Briefly, NC13 and picroside II, one of the catalpol derivatives isolated from NC13, displayed anti-inflammatory effects at 15 or 30 mg/kg oral administration without reported toxicity, and thus, mice were fed 20 mg/kg of either NC13 or Vms mixed with a powdered chow diet during the experimental period. The doses of NC13 and Vms are equivalent to 97.29 mg/day for 60 kg human^19^. As 80–160 mg of an NC13 based drug (YPL) is administered to human subjects in a clinical trial in USA, our 20 mg/kg dose is considered to be physiologically relevant.

### Animal study

Animals were used in accordance with protocols approved by Institutional Animal Care and Use Committee of the Ulsan National Institute of Science and Technology (UNISTIACUC-20-07). In our previous study, we developed the MMTV-FP635 mouse model for whole-body imaging of mammary tumor progression^20^. In this model, the infrared-fluorescence protein (FP635) is from mammary epithelial cells, specifically expressed under the control of a MMTV promoter. By crossing with MMTV-PyMT mice (MMTV-PyMT/FP635), we can monitor tumor growth and metastasis via whole-body fluorescent scanner, longitudinally without sacrificing the mice. For PyMT/FP635 mouse model, 8-week-old female mice received intraperitoneal injection of cisplatin, dissolved in PBS and sonicated for 3 min before use. For the first week, 2.5 mg/kg of cisplatin was administered and 1.5 mg/kg of cisplatin for the rest of the weeks. Mice were fed powdered chow diet mixed with either NC13 or Vms (20 mg/kg) during the experimental period. Tumor growth was monitored every week using an in vivo fluorescence imaging system (Bruker, Germany). After 10 weeks of cisplatin administration, animals were sacrificed, and lung and tumor samples were harvested for further experiments. In a tumor allograft model with BALB/C mouse, wild-type 9-week-old male mice were fed powdered chow diets mixed with either NC13 or Vms (20 mg/kg) during the experimental period. After 1 week of NC13 or Vms pre-treatment, 0.5 × 10^5^ of 4 T-1 cells were subcutaneously injected into the mammary fat pad. When the tumors reached approximately 100 mm^3^, 2.5 mg/kg of cisplatin was intraperitoneally injected twice in a week. Tumor growth was measured using digital caliper twice in a week, and after 6 weeks of cisplatin administration, animals were sacrificed, and lung and tumor samples were harvested for further experiments.

### Tissue staining and immunohistochemistry

Tissue samples were excised and fixed in buffered 10% formalin for 48 h. After dehydration and paraffin embedding, each tissue section was subjected to staining with hematoxylin and eosin (H&E) or was further processed for immunohistochemistry. After antigen unmasking with a citrate-based buffer and hydrogen peroxide treatment, sections were incubated with primary antibodies against Ki-67, followed by Biotin-rabbit IgG and streptavidin-HRP. Images were acquired with an Olympus FSX100 inverted microscope.

### Data and statistical analysis

All the data were analyzed by using GraphPad Prism software (Version 7, USA) and presented as mean ± standard error of the mean. Statistical significance was evaluated by one-way analysis of variance followed by post-hoc Tukey’s tests for multiple comparison. *P*-values less than 0.05 were considered statistically significant difference.

## Results

### Isolation of Vms as one of the major bioactive components of NC13

**Figure 1:**
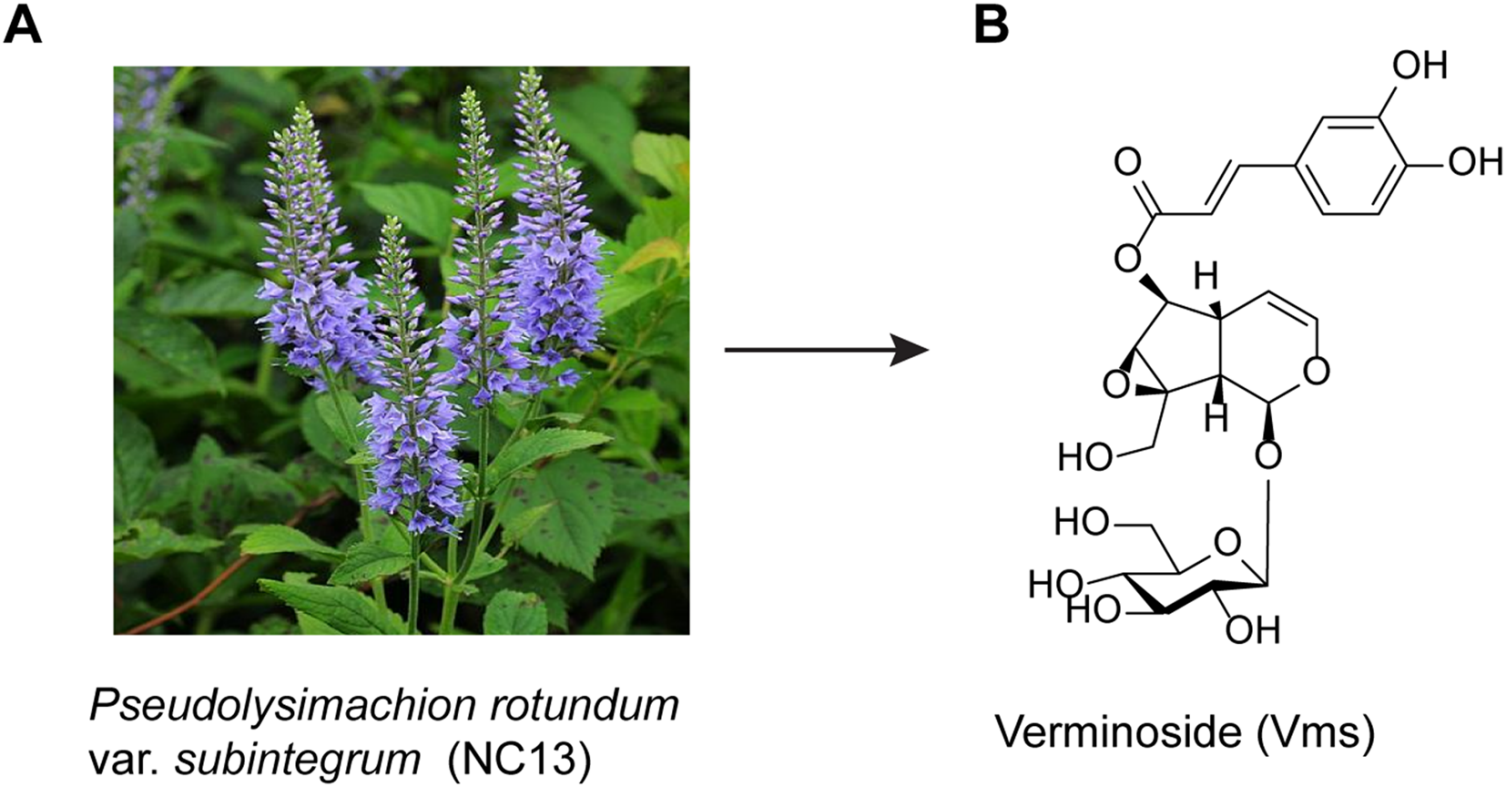
Chemical structure of verminoside (Vms) isolated from p. rotundum var. subintegrum (NC13). Also see Supplementary Fig. S5–4.

Vms is one of the major bioactive components of NC13 (voucher specimen: KRIB 0020697), and its chemical structure is displayed in Fig. 1 and Supplementary Fig. S1–4, along with a brief description of the isolation process.

### Combination therapy with either NC13 or Vms augments chemosensitivity to cisplatin

**Figure 2:**
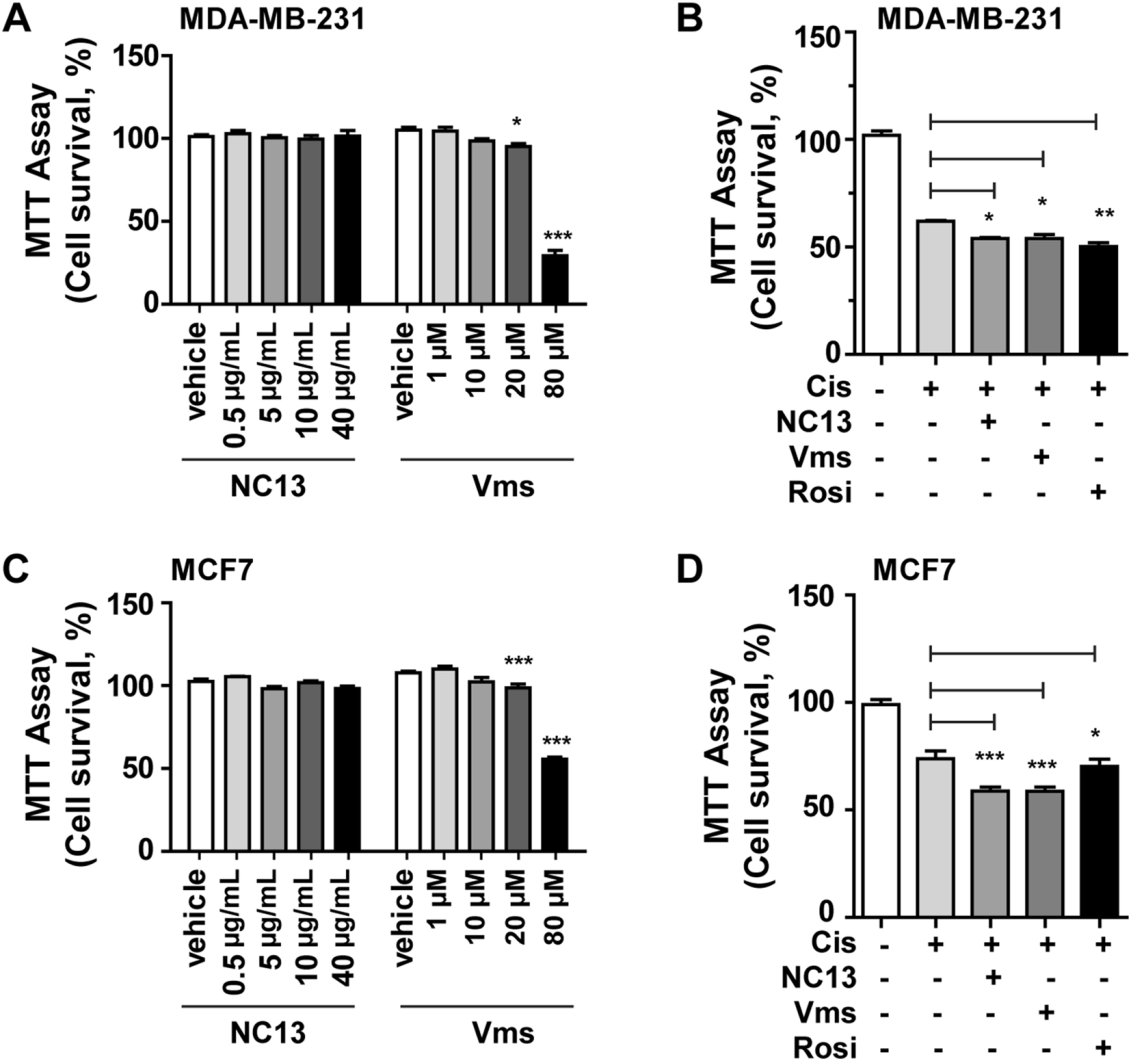
Chemoadjuvant effect of NC13 or Vms on the cellular survival of MDA-MB-231 and MCF7 cells. (**A**,**C**) MDA-MB-231 and MCF7 cells were treated with increasing concentrations of either NC13 or Vms for 48 h. (**B**,**D**) The cells were treated with either NC13 (5 µg/mL) or Vms (10 µM) in the presence of cisplatin. After 2 days, the MTT assay was performed to assess cellular viability. Rosiglitazone (Rosi, 20 μM) was used as a positive control. All data are presented as mean ± SEM. Statistical significance was evaluated by *one-way ANOVA*, followed by post hoc* tukey’s multiple comparison test*. ^*^P < 0.05, ^**^P < 0.01.

To evaluate the efficacy of NC13 and Vms as chemoadjuvants in cisplatin treatment, we first found the optimal concentrations at which there were no cellular toxicity effects. NC13 showed no cytotoxic effects on cellular proliferation below a concentration of 40 μg/mL, whereas Vms showed cytotoxic effects at a concentration of over 10 μM (= 5.244 μg/mL) (Fig. 2A,C). Therefore, we determined 5 μg/mL and 10 μM as the optimal concentrations for NC13 and Vms, respectively. The viability of breast cancer cell lines, such as MDA-MB-231 and MCF7, were determined at 48 h after NC13 or Vms treatment. Because most of the NC13 and Vms-induced synergistic effects on cisplatin monotherapy were found to be exerted at 48 h after drug treatment based on the CompuSyn analysis (Supplementary Fig. S5), we used these selected dosages and time for the in vitro experiments.

To investigate whether either NC13 or Vms had chemoadjuvant effects in cisplatin monotherapy, MDA-MB-231 and MCF7 cells were treated with NC13 or Vms in the presence of cisplatin, and cell survival was determined using the MTT assay. Optimal dosages of cisplatin were also determined by CompuSyn analysis, considering IC_50_ values of cisplatin for each breast cancer cell line (Supplementary Fig. S5). In the MDA-MB-231 cells, treatment with NC13 or Vms in combination with cisplatin significantly suppressed cell survival compared with the effect of cisplatin alone (Fig. 2B). Similarly, for the MCF7 cells, the combination treatment significantly augmented the efficacy of cisplatin for the suppression of cancer cell survival (Fig. 2D). Rosiglitazone (rosi), used as a positive control drug, has synergistic effects in cisplatin combination therapy; nevertheless, it has no cytotoxicity by Rosi alone likely with NC13 or Vms^21–23^. Notably, the efficacy of the combination of cisplatin with either NC13 or Vms was comparable or superior to that of the treatment with rosi. These results suggested that combination treatment of cisplatin with either NC13 or Vms sensitized breast cancer cells to the anti-cancer effects of cisplatin on cancer cell survival in vitro.

### NC13 or Vms attenuates the epithelial–mesenchymal transition (EMT) in breast cancer cells in vitro

It is well known that the EMT, in which cancer cells lose their epithelial properties and adopt mesenchymal characteristics, leads to reorganization of the actin cytoskeleton, decreased cell-to-cell contact, and loss of cell polarity^24^. As a result, invasive and metastatic activities increase in cancer cells. Beyond the cellular invasion and metastasis, multiple studies have suggested that sensitivity to chemotherapeutic drugs is closely associated with the EMT of cancer cells^25^. EMT contributes to chemoresistance in several ways, including EMT-related microRNA and cytokine production^24^, and EMT-driven cancer cell stemness is one of the most well-known mechanisms.

**Figure 3:**
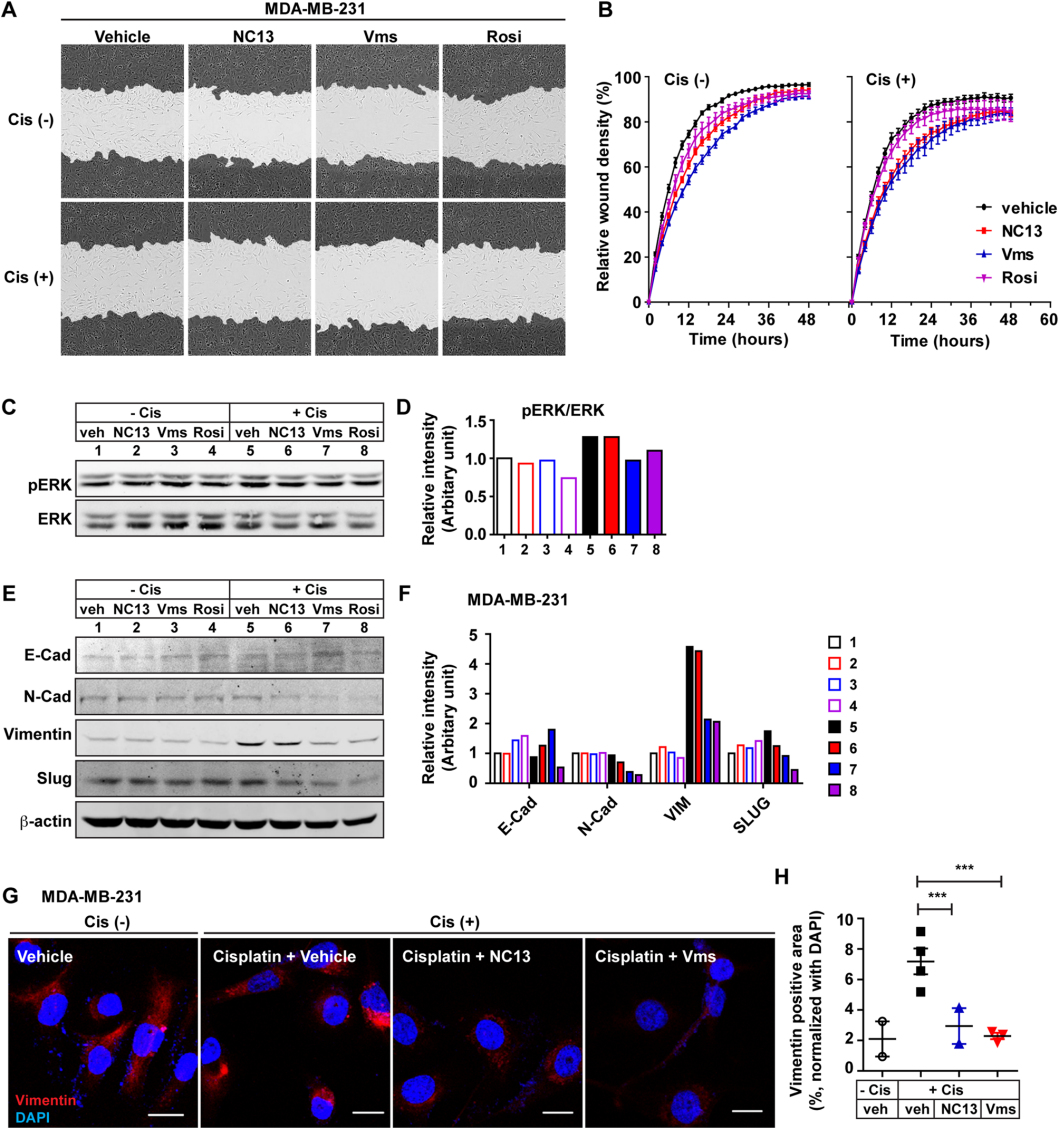
Chemoadjuvant effect of NC13 or Vms on the EMT in MDA-MB-231 breast cancer cells. (**A**) MDA-MB-231 cells were scratched, and representative images were taken after 24 h of either NC13 (5 μg/ml) or Vms (5 μg/ml, 9.53 μM) treatment in the presence or absence of cisplatin (20 μM). Rosi (10 μM) is used as a positive control. (**B**) The wound density was quantified in the indicated treatment. (**C**) Western blotting analysis was performed to determine ERK phosphorylation in indicated groups, and (**D**) their band intensity was quantified. (**E**) The protein levels of EMT markers, including E-Cadherin, N-Cadherin, Vimentin, and Slug were determined, and (**F**) their band intensity was quantified. (**G**) Immunofluorescence staining of vimentin and (**H**) the signal intensity was quantified for the indicated treatments (scale bar = 20 μm). All data are presented as mean ± SEM. Statistical significance was evaluated by *one-way ANOVA*, followed by post hoc* tukey’s multiple comparison test*. ^*^P < 0.05, ^***^P < 0.001 vs. vehicle (− cis); ^#^P < 0.05, ^###^P < 0.001 vs. vehicle (+ cis). Uncropped blots are shown in the Supplementary information.

Therefore, we examined the effect of NC13 and Vms on the EMT process in breast cancer cells. As functional indices of EMT, we determined the cell migration ability of the MDA-MB-231 cells following treatment with either NC13 or Vms in the presence or absence of cisplatin. Both NC13- and Vms-treated cells displayed reduced migration ability compared with that of the control cells in the presence or absence of cisplatin, as determined by the cell migration assay in vitro (Fig. 3A,B). The extracellular signal-regulated kinase (ERK) pathways are well known to regulate various biological processes including proliferation, cell migration, and invasion in response to environmental stimuli such as chemotherapy^26,27^. Treatment of the MDA-MB-231 cells with cisplatin elevated the activity of the ERK signaling pathways compared to that in the vehicle group without cisplatin. However, the combination of cisplatin with NC13 and Vms showed no additional (or synergistic) effect on the cisplatin-induced ERK activation (Fig. 3C,D). This showed that NC13 or Vms has a limited effect on cisplatin-mediated ERK activation in the MDA-MB-231 cells. To further explore whether the EMT processes were altered by the adjuvant therapy, the levels of EMT markers such as vimentin, snail, slug, N-cadherin, and E-cadherin were analyzed. Overall, cisplatin treatment increased the levels of mesenchymal cell markers such as N-cadherin, vimentin, snail, and slug compared to that in control groups (no cisplatin groups) in the MDA-MB-231 cells (Fig. 3E,F). Furthermore, the levels of these mesenchymal cell markers significantly decreased by the combination of cisplatin with NC13 and Vms, comparable to the levels after treatment with Rosi in combination with cisplatin, used as a positive control (Fig. 3E,F). The level of E-cadherin, an epithelial cell marker, was not affected by the combination of cisplatin with NC13 and Vms compared with that in the control groups (no cisplatin groups) (Fig. 3E,F). These results indicated that combination of both NC13 and Vms with cisplatin may contribute to altering the levels of mesenchymal marker proteins rather than activating upstream signaling pathways such as the ERK. This was further validated with immunofluorescence staining of vimentin in the MDA-MB-231 cells, which strongly suggested that combined treatment with either NC13 or Vms significantly attenuated the cisplatin-associated increase of the EMT process (Fig. 3G,H).

**Figure 4:**
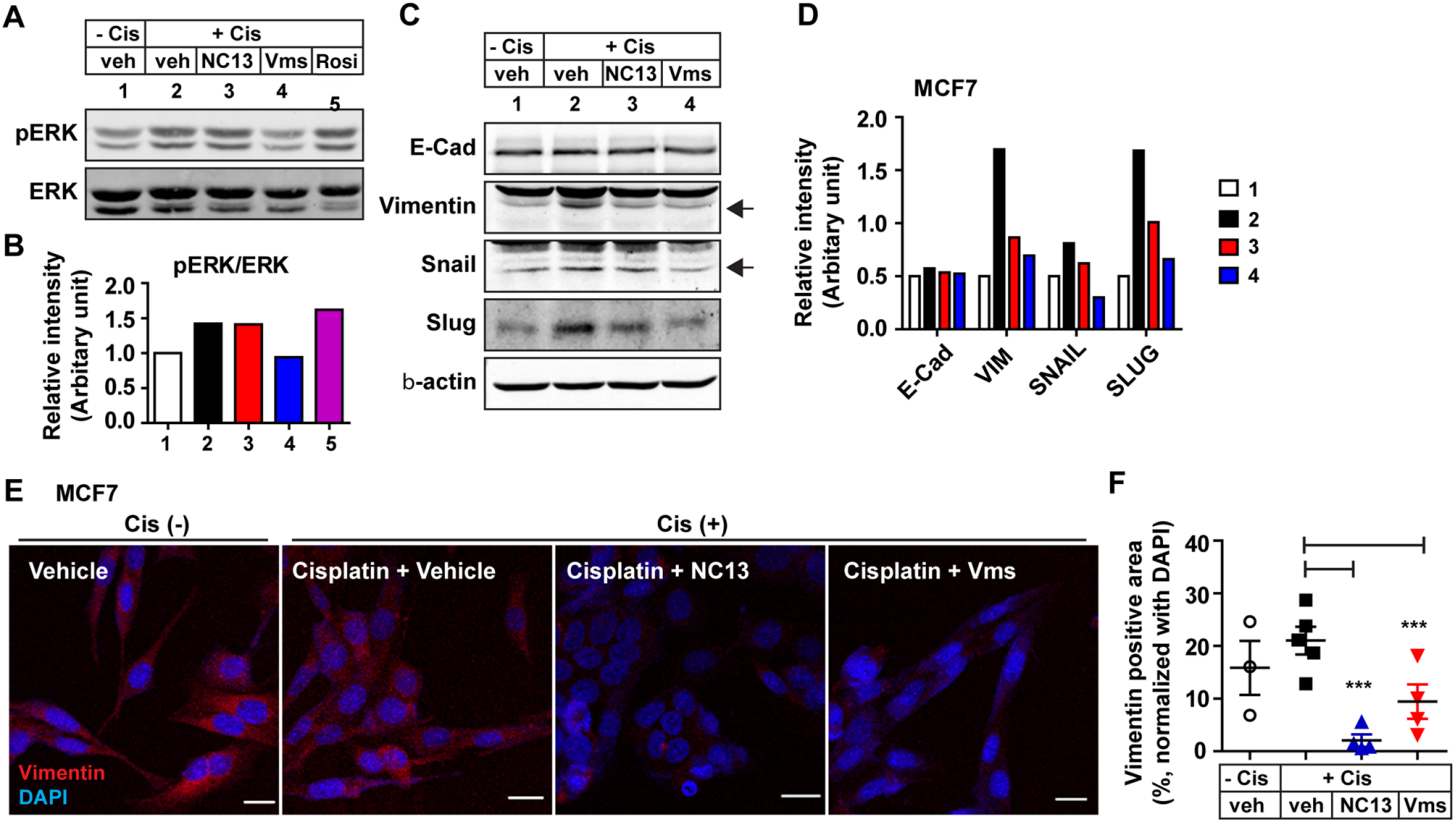
Chemoadjuvant effect of NC13 or Vms in cisplatin treatment on the suppression of the EMT in MCF7 breast cancer cells. (**A**) Western blotting analysis was performed to determine ERK phosphorylation and (**B**) the band intensity was quantified. (**C**) The protein levels of EMT markers, including E-Cadherin, Vimentin, Snail, and Slug were determined by western blots, and (**D**) the band intensity was quantified. (**E**) Immunofluorescence staining of vimentin and (**F**) the signal intensity was quantified for the indicated treatments (scale bar = 20 μm). All data are presented as mean ± SEM. Statistical significance was evaluated by *one-way ANOVA*, followed by post hoc* tukey’s multiple comparison test*. ^***^P < 0.001. Uncropped blots are shown in the Supplementary information.

Consistently, the ERK signaling pathways were not altered or even slightly reduced in the NC13 and Vms groups, respectively, compared to the case in the vehicle group in the presence of cisplatin (Fig. 4A,B). In addition, the protein levels of EMT markers, particularly vimentin and snail, significantly decreased by NC13 and Vms treatment in combination with cisplatin compared with those in the cisplatin control group (Fig. 4C,D). Immunostaining of vimentin in the MCF7 cells clearly indicated that the EMT was suppressed by NC13 and Vms in the cisplatin treatment group compared with that in the vehicle group (Fig. 4E,F). Collectively, these data suggested that the combination treatment of cisplatin with NC13 or Vms enhanced the suppression of the EMT phenotype in both MDA-MB-231 and MCF7 cells, partly through suppression of the EMT process.

### Combination of NC13 or Vms with cisplatin attenuates metastatic growth in MMTV-PyMT mammary tumor mice in vivo

**Figure 5:**
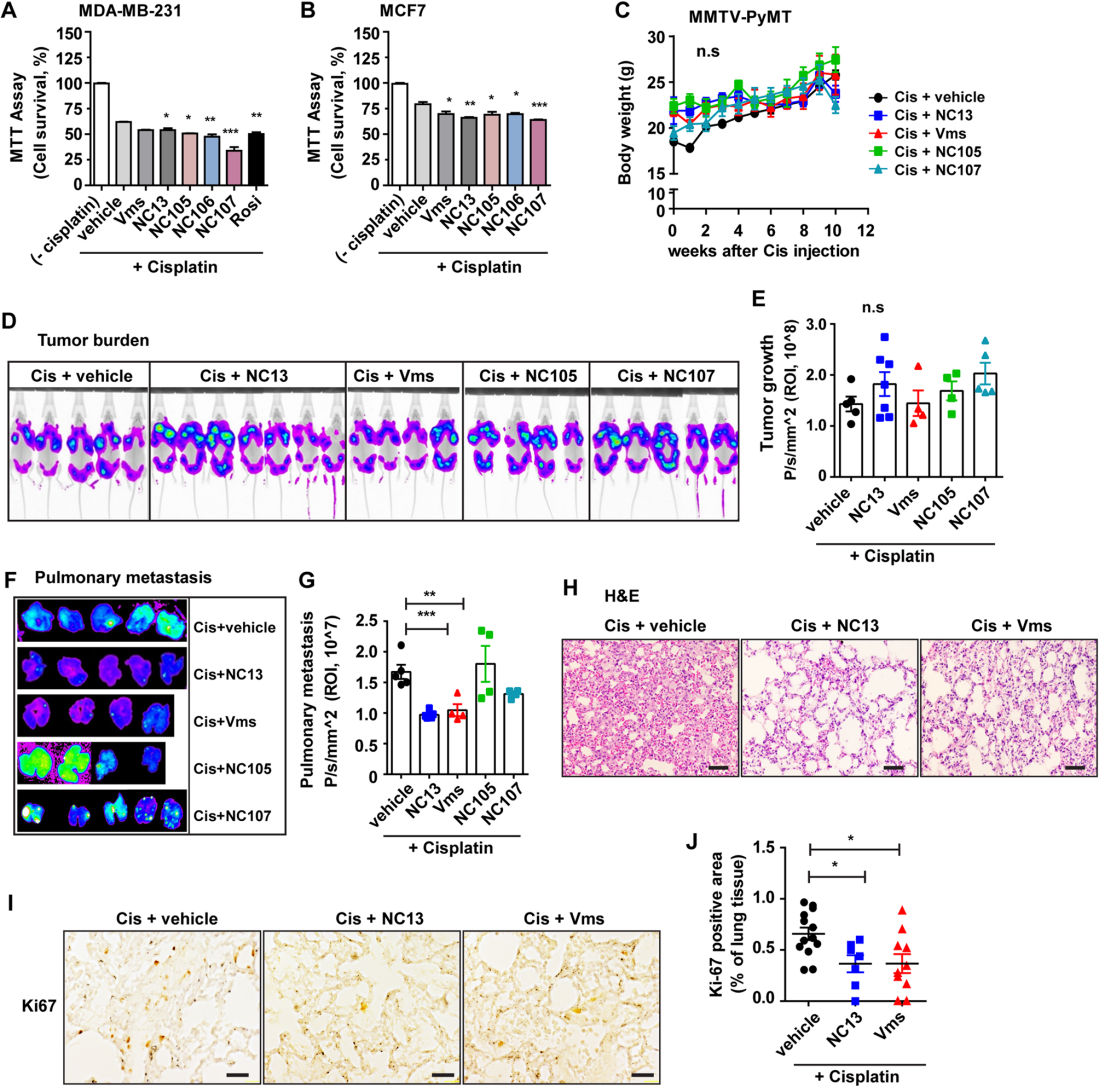
Anti-metastatic effects of NC13 in combination with cisplatin are mostly mediated by Vms in the MMTV-PyMT breast cancer mouse model. (**A**,**B**) The breast cancer cells were treated with either NC13 or its isolated components including Vms (5 µg/mL), NC105—Picroside II (5 µg/mL), NC106—Isovanilly catalpol (5 µg/mL), and NC107—6-o-veratroyl catalpol (5 µg/mL) in the presence of cisplatin (10 μM). After 2-days, an MTT assay was performed to assess cellular viability. Rosi (10 μM) was used as a positive control. (**C**–**J**) MMTV-PyMT mice were injected with Cisplatin and fed a powdered diet mixed with either NC13 or its isolated bioactive components including Vms, NC105 and NC107 during the period of tumor progression. (**C**) Body weight was measured. (**D**) Fluorescent images of tumor burden in whole body were presented and (**E**) the tumor burden in each group was quantified. (**F**) Representative fluorescent images of lung metastatic burden are shown and (**G**) quantification of metastatic burden. (**H**) H&E staining and (**I**) immunohistochemistry of ki-67 were performed on lung tissues and (**J**) their areas were quantified (scale bar = 20 μm). All data are presented as the mean ± SEM. Statistical significance was evaluated by *one-way ANOVA*, followed by post hoc* tukey’s multiple comparison test*. ^*^P < 0.05, ^**^P < 0.01, ^***^P < 0.001.

As we have isolated several major components from NC13 plant extracts including Vms, picroside II (NC105), isovanilly catalpol (NC106), and 6-*O*-veratroyl catalpol (NC107) (Supplementary Fig. S1–4), these components were also tested for their chemoadjuvant effects on cisplatin. MDA-MB-231 and MCF7 breast cancer cells were treated with NC13 or its major components in combination with cisplatin, and subsequently, the cancer cell survival rate was determined (Fig. 5A,B). Similar to NC13 and Vms, treatment of NC105, NC106, and NC107 in combination with cisplatin significantly suppressed cell survival compared to that of cisplatin alone (Fig. 5A,B). Therefore, we pursued analyzing the chemoadjuvant effects of NC13 and its major components such as Vms, NC105, and NC107 in in vivo settings. NC106 was not included in this setting due to its mild adjuvant effects on the in vivo pilot study. To evaluate the chemoadjuvant effect of NC13 or its major components in combination with cisplatin in vivo animal model, the mouse mammary tumor virus-polyoma middle tumor-antigen (MMTV-PyMT) mice crossed with MMTV-infrared fluorescent protein (FP635) mice were utilized, which allow the visualization of tumor growth via fluorescent images longitudinally^22^. In combination with NC13 or its major components including Vms, NC105, and NC107, cisplatin treatment did not result in significant differences in body weight change, an index of drug toxicity (Fig. 5C). We further confirmed that cisplatin combination treatment with NC13 nor Vms did not induce adverse cytotoxic side effects in an in vivo experimental setting as determined by hepatotoxicity such as ALT, AST activities and histological assessment of liver tissue sections (Supplementary Fig. S6). Moreover, the chemoadjuvant activity of NC13 and its major components on the primary tumor growth was limited as compared to that in cisplatin alone (Fig. 5D,E). Next, we subsequently analyzed pulmonary metastasis as determined by in vivo fluorescent imaging (Fig. 5F). In combination with NC13 or Vms, cisplatin treatment significantly reduced metastatic growth compared with cisplatin alone or in combination with either NC105 or NC107 treatment (Fig. 5F,G), suggesting that the chemoadjuvant activity of NC13 in suppression of metastatic growth is mostly mediated by Vms, rather than NC105 or NC107, in the MMTV-PyMT settings. Accordingly, H&E staining and immunohistochemical staining for ki-67 indicated that the combination treatment of cisplatin with NC13 or Vms significantly inhibited pulmonary metastasis in a MMTV-PyMT mammary tumor mouse model (Fig. 5H–J).

### Combination of NC13 or Vms with cisplatin attenuates metastatic growth of 4T-1 breast cancer cells in vivo

We further confirmed the chemoadjuvant activity of NC13 or Vms in the suppression of tumor metastasis by using a 4T-1 tumor allograft model. The 4T-1 mammary carcinoma cell line was originally developed by Miller and his coworkers; it is derived from the mammary tumors of BALB/C mice. As this cancer cell is transplantable to mammary glands, highly aggressive, and able to spontaneously metastasize to other organs, the 4T-1 tumor implantation model is suitable for a human breast cancer study.

**Figure 6:**
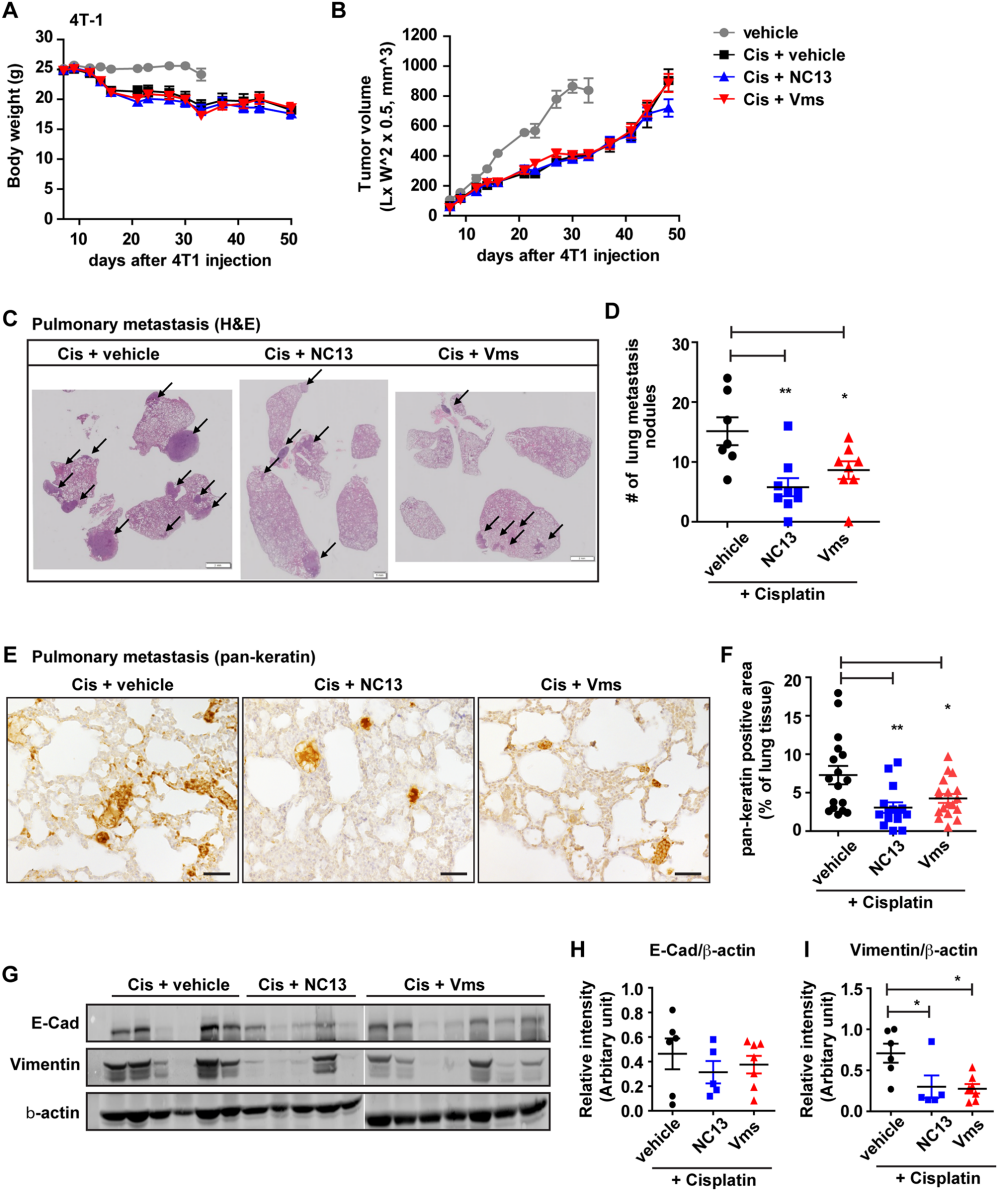
Chemoadjuvant effect of NC13 or Vms in cisplatin treatment on the 4T-1 breast cancer cells allograft model. (**A**) Body weight of the BALB/C mice administered cisplatin with either NC13 or Vms was measured compared to the vehicle group (− cisplatin). (**B**) Tumor volumes were measured in the indicated groups. (**C**) Representative H&E staining of the lung images were shown, and (**D**) the number of lung metastatic nodules were quantified. Arrowheads indicate tumor metastatic nodules. (**E**) Immunohistochemistry of pan-keratin were performed on lung tissues and (**F**) their area was quantified. Scale bar = 50 μm. (**G**) Protein levels of vimentin and E-cadherin in tumor tissue were determined by western blotting, and (**H–I**) quantification of band intensity from the indicated mice groups. Protein samples for the Cis + Vms group were blotted in a separated gel and analyzed with the same exposure done for the other blots including Cis + vehicle and Cis + NC13 groups. All data are presented as mean ± SEM. Statistical significance was evaluated by *one-way ANOVA*, followed by post hoc* tukey’s multiple comparison test*. ^*^P < 0.05, ^**^P < 0.01, ^***^P < 0.001. Uncropped blots are shown in the Supplementary information.

Six weeks after 4T-1 breast cancer cell implantation into the mammary glands, the mice were sacrificed, and the lung tissues were harvested. The cisplatin-treated groups showed lower body weights than the vehicle group due to the drug toxicity of cisplatin, while the combination with NC13 or Vms in cisplatin did not affect additional body weight differences compared to cisplatin alone (Fig. 6A), which was consistent with MMTV-PyMT mice. Similarly, there were no significant differences in the primary tumor growth from combination treatment with NC13 or Vms in cisplatin compared with those treated with cisplatin alone (Fig. 6B). Consistent with the results from the MMTV-PyMT models, the adjuvant chemotherapy of NC13 or Vms with cisplatin significantly suppressed the metastatic growth compared with the cisplatin control group, as determined by the numbers of metastatic nodules stained with H&E (Fig. 6C,D) and pan-keratin (Fig. 6E,F). To further assess the expression of EMT markers in tumor tissues, a western blotting analysis was performed. The mesenchymal marker vimentin was downregulated in tumors from mice treated with the combination of cisplatin with NC13 or Vms compared with mice treated with cisplatin, whereas there was no significant change in E-cadherin expression (Fig. 6G–I). Collectively, these results strongly suggested that the combination of NC13 or Vms with cisplatin inhibited the EMT in tumor cells, particularly through the downregulation of vimentin resulting in suppression of pulmonary metastasis in vivo.

## Discussion

Treatment with chemotherapeutic agents often fails in causing successful cancer remission when used in monotherapy. Hence, it is widely accepted to combine two or more chemotherapeutic drugs to achieve better outcomes, referred to as multidrug treatment. During the past few decades, studies have focused on the molecular and pharmacological factors that affect the efficacy of drug combinations, such as cellular pathways, drug interactions, optimal administration ratios, side effects, and consequently have given us a better understanding of multidrug combinations^28^. This progress leads us to develop combination therapeutic regimens that dramatically improve outcomes for cancer patients. For example, nowadays, a combination of cisplatin with docetaxel or gemcitabine is a first-line chemotherapeutic regimen for metastatic breast cancer patients^29,30^. Chemo-adjuvant therapy, on the other hand, is a combinatorial approach which employs the different forms of treatment such as chemotherapy with radiation therapy, instead of multiple anti-cancer drugs^31,32^. According to the National Cancer Institute (NCI) guideline, adjuvant therapy is defined as an additional cancer treatment given after the primary treatment to lower the risk that the cancer will recur. Recent advances in adjuvant therapies include combining newer agents with conventional chemotherapy. Among the various kinds of regimens, natural compounds are reported to have positive adjunctive effects in combination with chemo- or radiotherapy^33^. This natural compound based chemoadjuvant therapy could augment cytotoxicity to cancer cells and have additional effects on the immune response to tumors or the tumor microenvironment^34^.

In this study, we aimed to develop the natural compounds used for chemoadjuvants in conventional chemotherapy. We presented the first evidence of the therapeutic potential of natural products NC13 and its major component, Vms as chemoadjuvants in cisplatin monotherapy, and validated their anti-metastatic activity in vitro and in vivo animal models. The screening criteria of these compounds were based on their cytotoxic effects on breast cancer cells; thus, they have no cytotoxic effects by themselves, but they convey a synergistic (or additive) cytotoxic effect on cisplatin monotherapy. Based on this screening criteria, NC13 was firstly identified and subsequently Vms was further isolated as the major components derived from it. In the in vitro settings, NC13 and Vms exerted synergistic effects on cisplatin-induced suppression of cell growth and cell migration. Interestingly, these chemoadjuvants combined with cisplatin showed only a mild effect on primary tumor growth but they significantly suppressed metastatic growth in tumor-bearing mice including MMTV-PyMT and 4T-1 allograft models in vivo. We confirmed the potent anti-metastatic effects of NC13 combination in cisplatin with an additional in vivo model, in which 4T-1 cancer cells were intravenously injected into BALB/C mice and the pulmonary metastatic growth was assessed; thus the ability for metastatic growth of circulating cancer cells would be assessed (Supplementary Fig. S7). In this model, NC13 showed no effects per se on the metastatic growth of cancer cells likely vehicle; but NC13 in combination with cisplatin significantly suppressed pulmonary metastasis compared to cisplatin monotherapy (Supplementary Fig. S7B,C). These phenomena were also true for other chemoadjuvants such as Rosi and metformin, which have no cytotoxic effects on cancer cells, but they confer synergistic effects on conventional chemotherapy^23,35^. Molecular mechanisms underlying the Rosi-induced synergistic effects on cancer cell death are still largely elusive. Among the various major components isolated from NC13 including picroside II, isovanilly catalpol, 6-*O*-veratroyl catalpol, and Vms, the most significant anti-metastatic effect of cisplatin treatment in vivo was found in combination with Vms, revealing that Vms could mostly account for the NC13-mediated chemoadjuvant effects in cisplatin.

**Figure 7:**
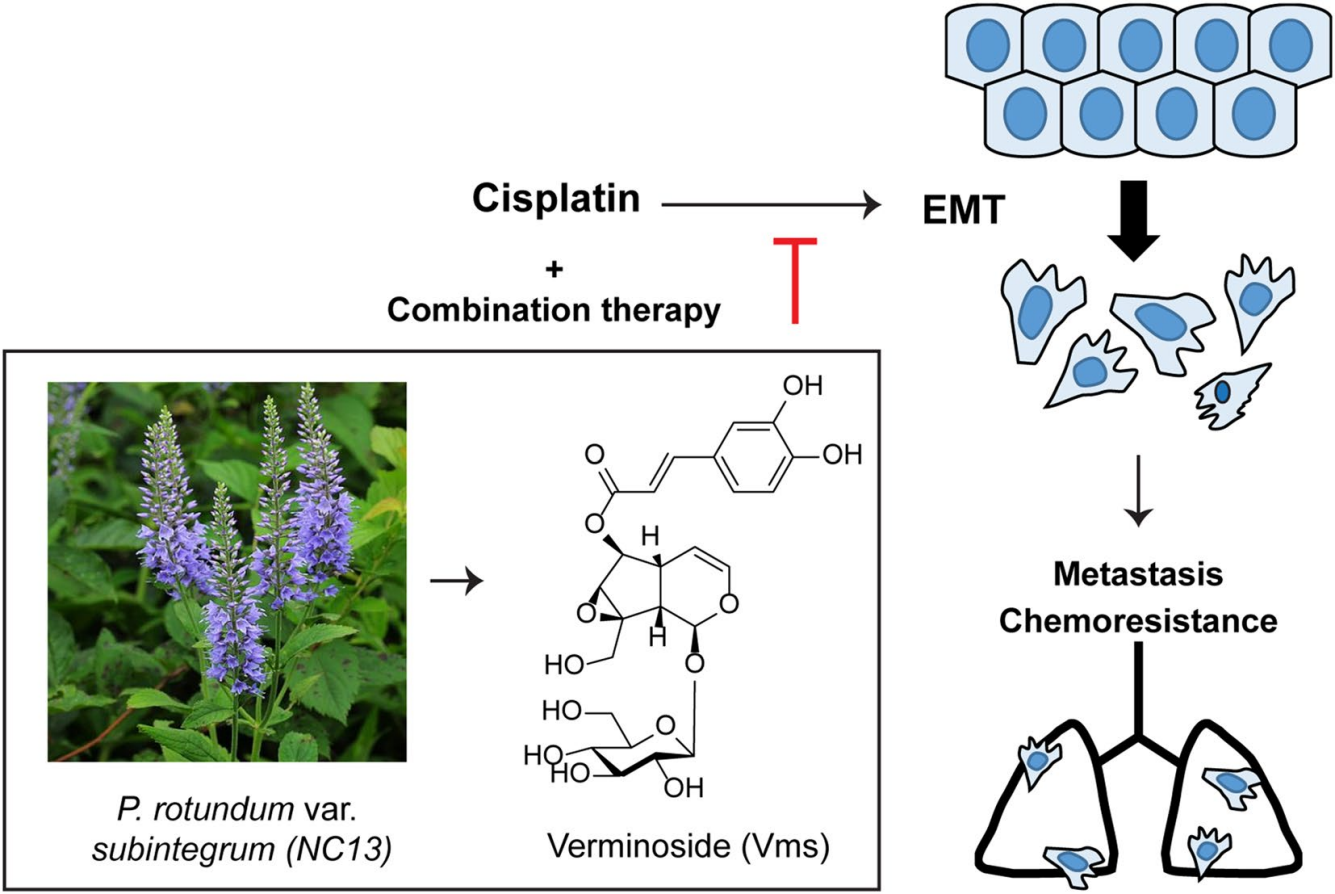
Schematic view of chemoadjuvant effect of both NC13 and Vms in cisplatin treatment in metastatic breast cancer. Verminoside (Vms) isolated from *Pseudolysimachion rotundum var. subintegrum* (NC13) sensitizes cisplatin effects in breast cancer. Combined administration of cisplatin with either Vms or NC13 sensitizes cisplatin treatment, and further suppresses cancer cell migration through down-regulation of the EMT process leading to significant inhibition of pulmonary metastasis and chemoresistance.

As tumor metastasis accounts for over 90% of mortalities in solid cancers, development of targeted therapeutic agents against cancer invasion or metastasis is important for drug discovery^36^. Several studies suggested the potential of anti-metastatic agents in the breast cancer preclinical models^37–39^, moreover, sacituzumab and govitecan-hziy for the patients of metastatic breast cancer are in phase III clinical trials^40^. Here, we suggested that Vms, isolated from NC13, gives a synergistic effect on cisplatin-based chemotherapy, particularly at the level of metastatic growth, and this is partly through the suppression of cisplatin-induced EMT processes (Fig. 7). Various applications of combination therapy have been developed to overcome the failure of conventional chemotherapy, and our study strongly suggested the efficacy of natural product NC13 and its isolated bioactive component, Vms as resources to be developed as chemoadjuvants and potentially as anti-metastatic agents in conventional chemotherapy; particularly, for metastatic breast cancer patients.

## Supplementary information

Supplementary Information.
