# Supplementary material for: Verminoside from Pseudolysimachion rotundum var. subintegrum sensitizes cisplatin-resistant cancer cells and suppresses metastatic growth of human breast cancer
Source: Sci Rep. 2020 Nov 23;10:20337. doi: 10.1038/s41598-020-77401-7 (PMC7683595; doi:10.1038/s41598-020-77401-7)
Supplement: Supplementary file 1 — Supplementary Information. [file 41598_2020_77401_MOESM1_ESM.docx]

**Supporting Information**

**Verminoside from *Pseudolysimachion rotundum* var. *subintegrum* sensitizes cisplatin-resistant cancer cells and suppresses metastatic growth of human breast cancer**

Changhu Lee^†,§^, Hyung Won Ryu^‡,§^, Sahee Kim^†^, Min Kim^†^, Sei-Ryang Oh^‡^, Kyung-Seop Ahn^‡^, and Jiyoung Park^†^*

^†^Department of Biological Sciences, Ulsan National Institute of Science and Technology (UNIST), Ulsan 44919, Republic of Korea

^‡^Natural Medicine Research Center, Korea Research Institute of Bioscience and Biotechnology (KRIBB), Cheong-ju si, Chungcheongbuk−do, 28116, Republic of Korea

**Table of Contents**

**Figure S1.** Preparative HPLC fractionation of *P. rotundum* var. *subintegrum* extract 2

**Figure S2.** UPLC-PDA of fractions of *P. rotundum* var. *subintegrum* extract 3

**Figure S3.** ^1^H and ^13^C NMR spectrum of verminoside 4

**Figure S4.** UV, MS/MS, MS and HREIMS data of verminoside 5

**Figure S5.** Comupsyn analysis of the NC13 or Vms chemoadjuvant therapy 7

**Figure S6.** Assessment of drug toxicity in the *in vivo* mouse models 8

**Figure S7.** Chemoadjuvant effect of NC13 in cisplatin on the *in vivo* metastasis model 9

**Figure S8.** Uncropped blots corresponding to Figure 3C, E 7

**Figure S9.** Uncropped blots corresponding to Figure 4A, B 8

**Figure S10.** Uncropped blots corresponding to Figure 6G 9

**Experimental Section**

1D (^1^H, and ^13^C) NMR spectra were obtained on JEOL ECZ500R (^1^H NMR at 500 MHz, ^13^C NMR at 125 MHz, Tokyo, JP) using acetnone-*d*_6_ (Cambridge Isotope Laboratories, Andover, MA) as an NMR solvent and tetramethylsilane (TMS) as an internal standard. HRESIMS were measured on an ultraperformance liquid chromatography quadrupole time-of-flight mass spectrometer (UPLC-QTof-MS, Waters, Milford, MA, USA) in the negative-ion mode. Preparative HPLC was performed with a K-Prep LAB-300G instrument (YMC, Kyoto, Japan). Semiprepartive HPLC separation (Gilson, Middleton, WI, USA) is comprised of a standard binary pump (321 HPLC pump), UV/Vis detector (172 DAD), evaporative light-scattering detector (ELSD, Varian 380-LC), and injection modules (GX271 liquid handler).


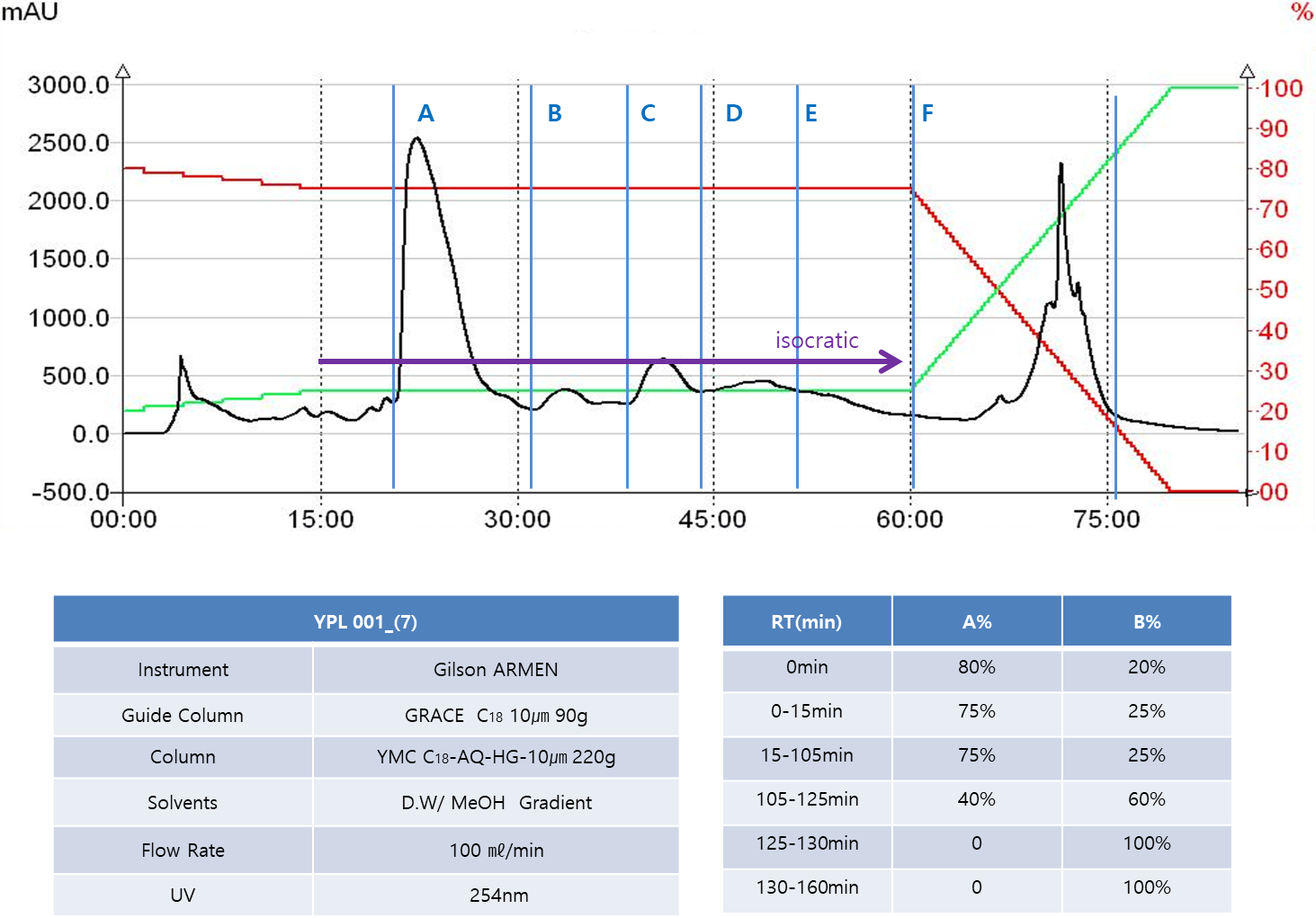


**Figure S1. Preparative HPLC fractionation of *P. rotundum* var. *subintegrum* extract.**


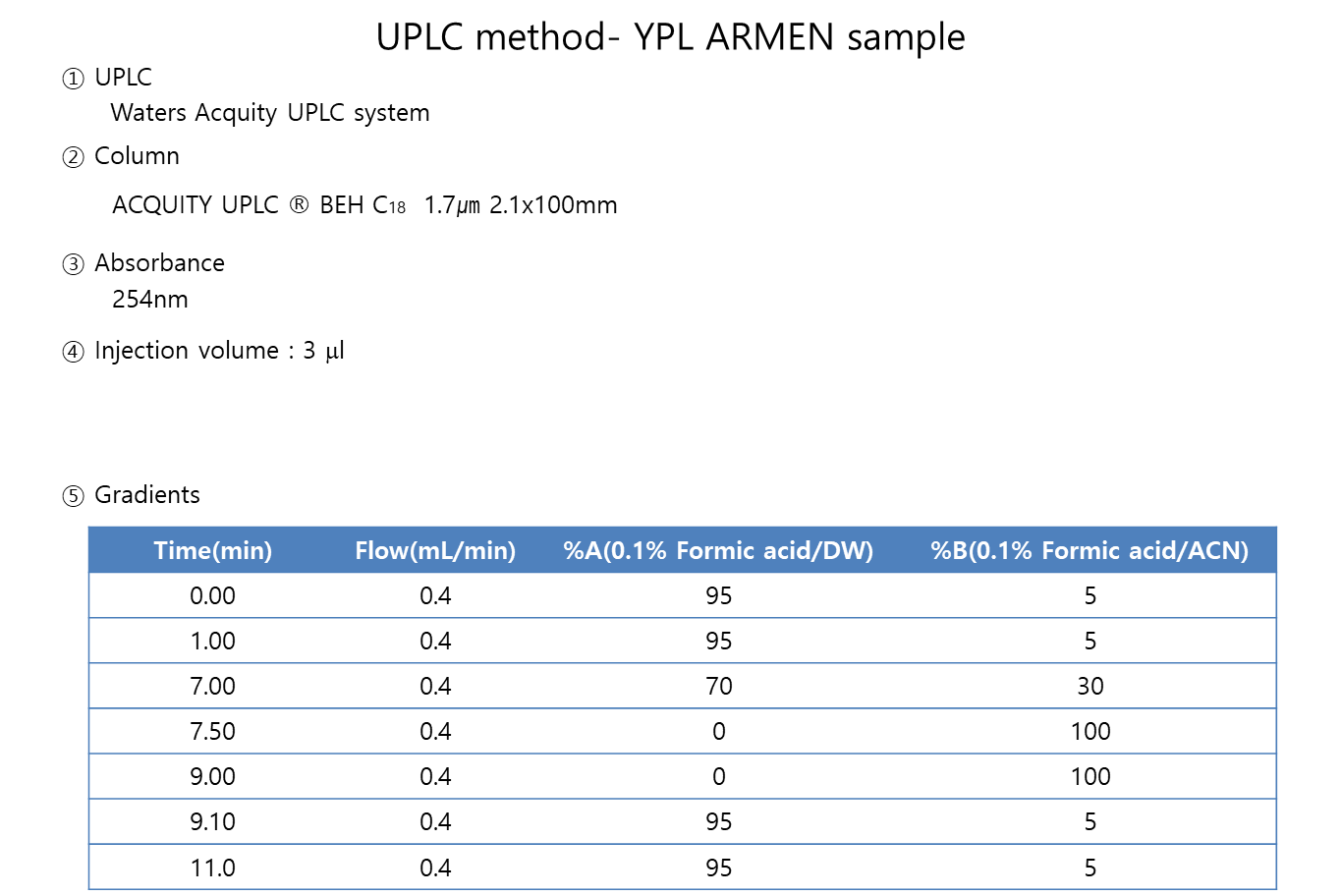

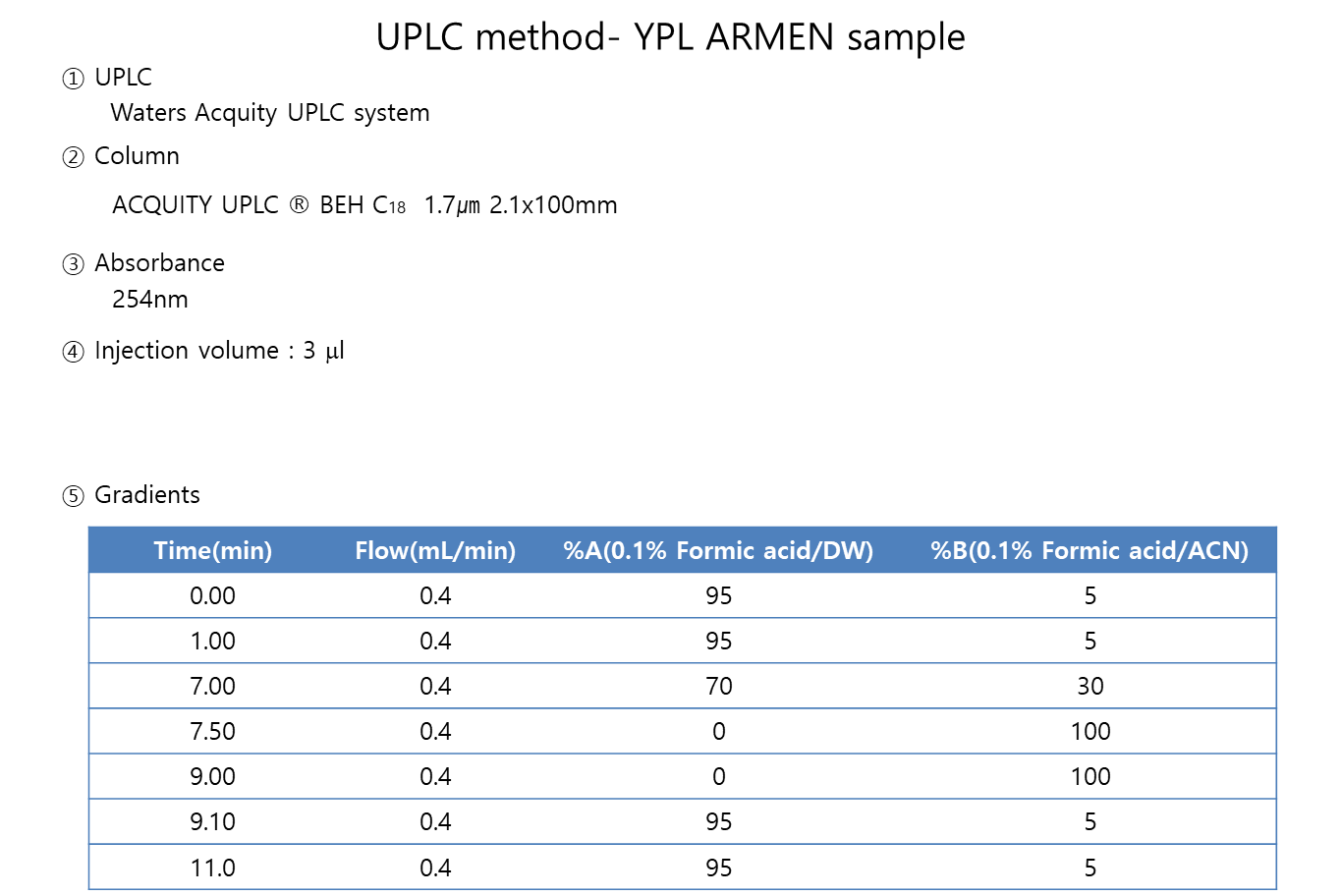

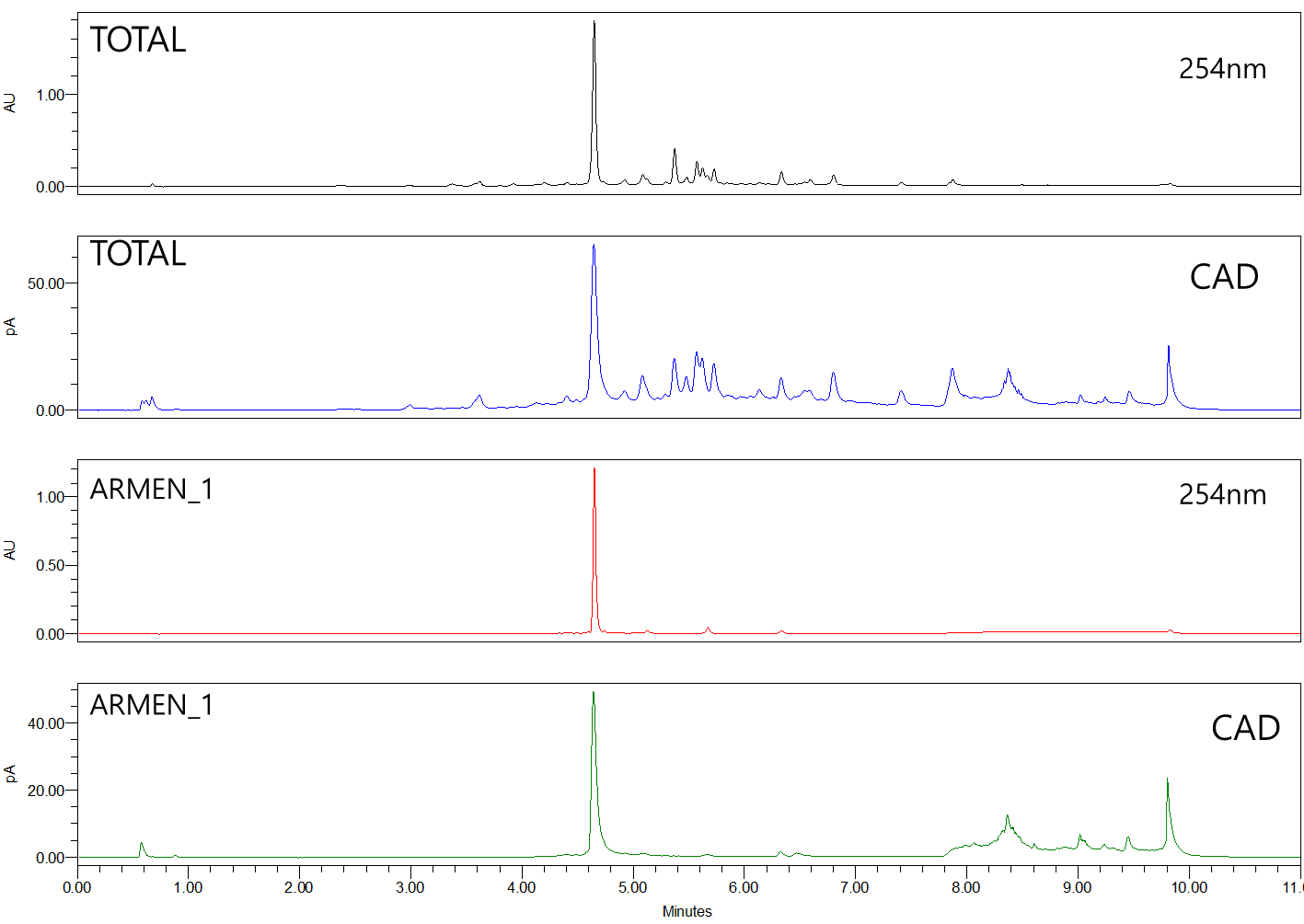

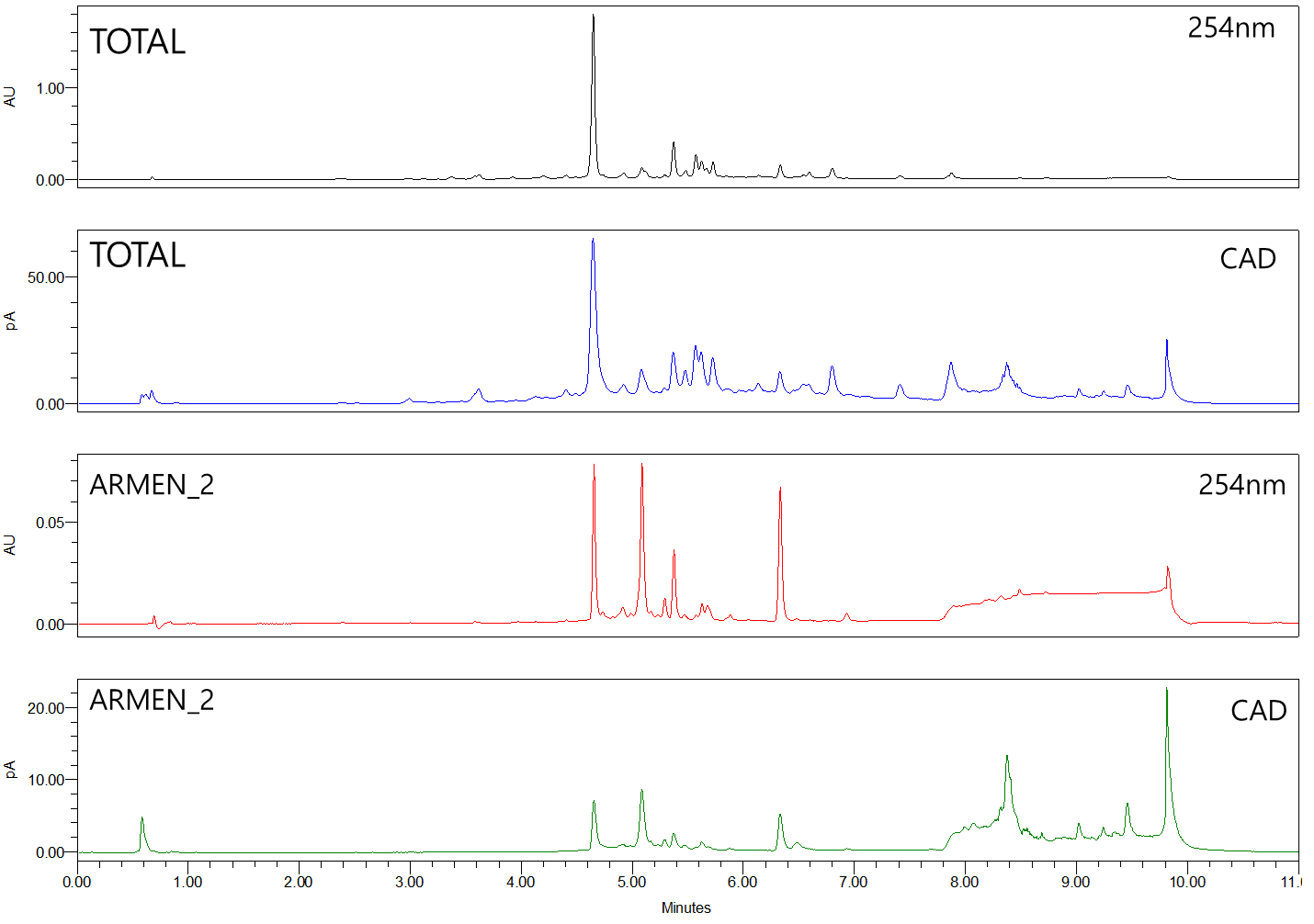


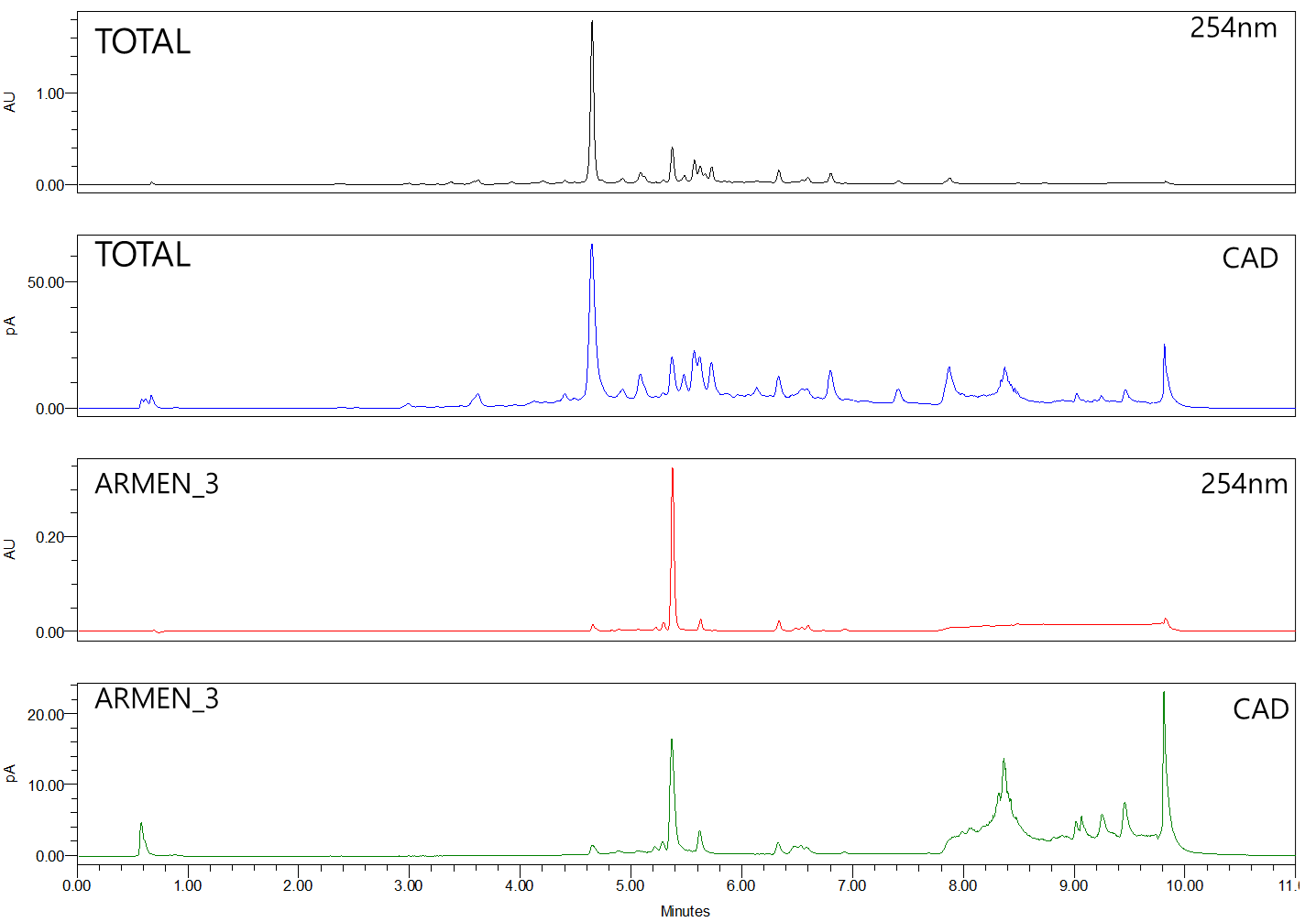

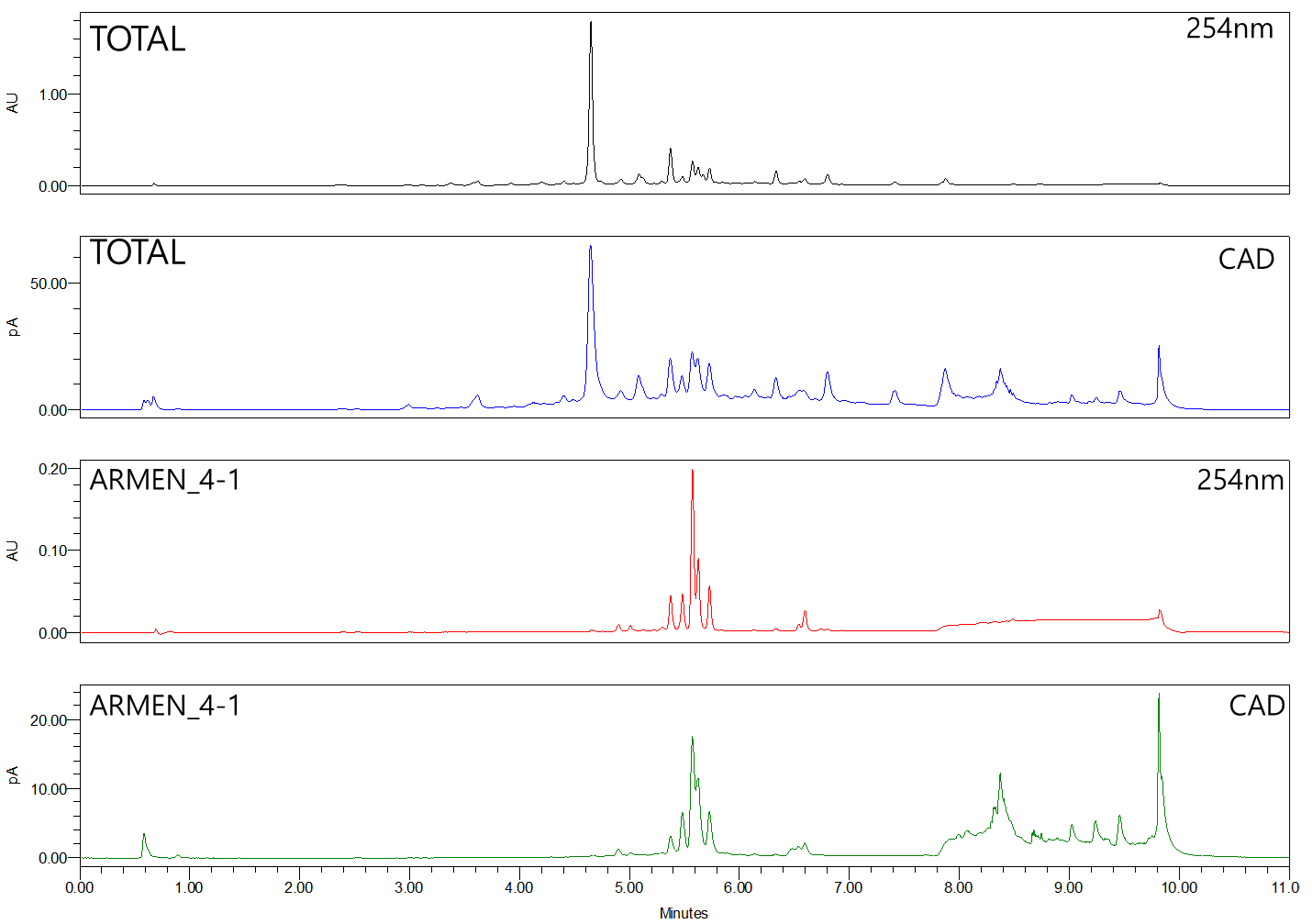


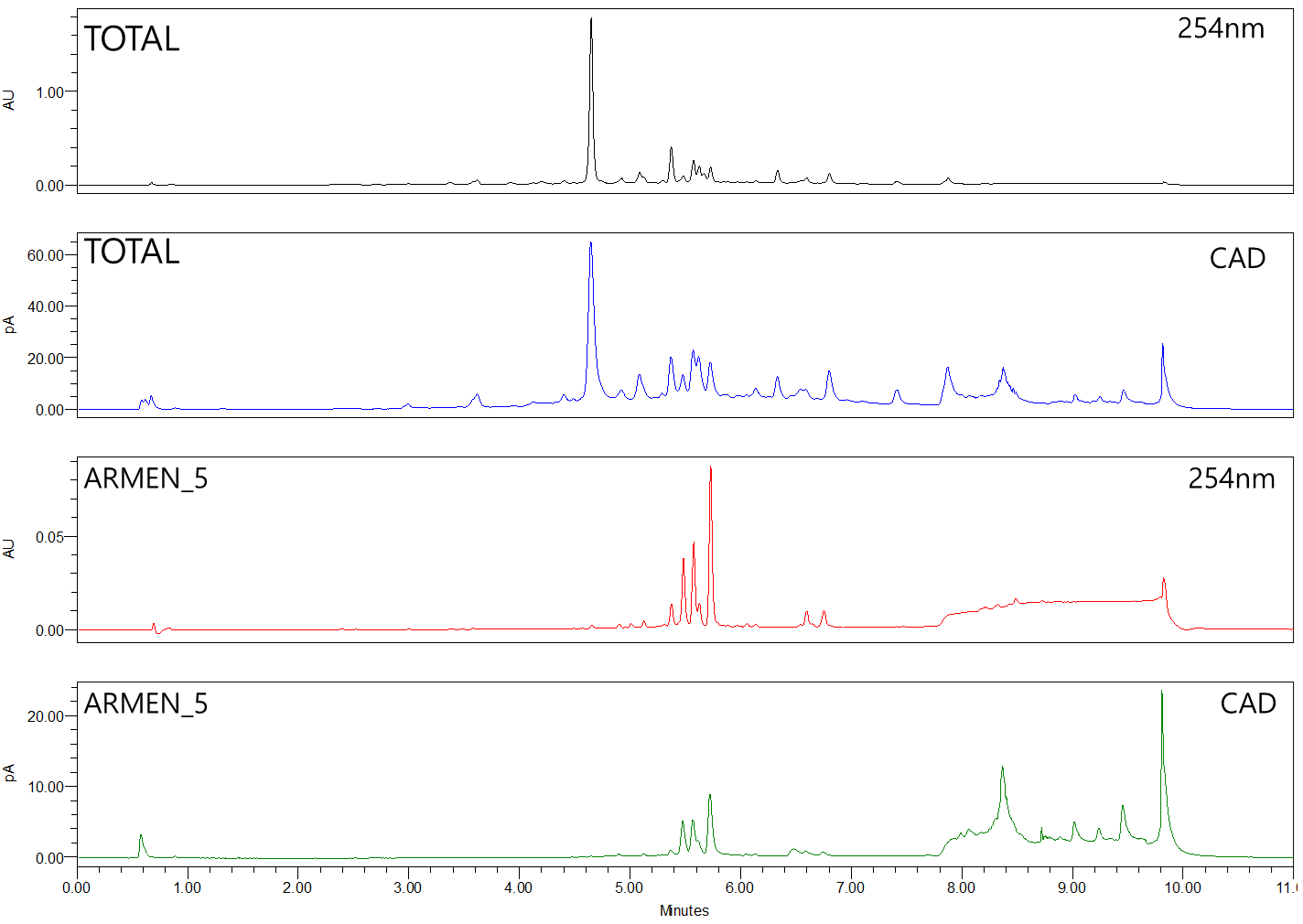

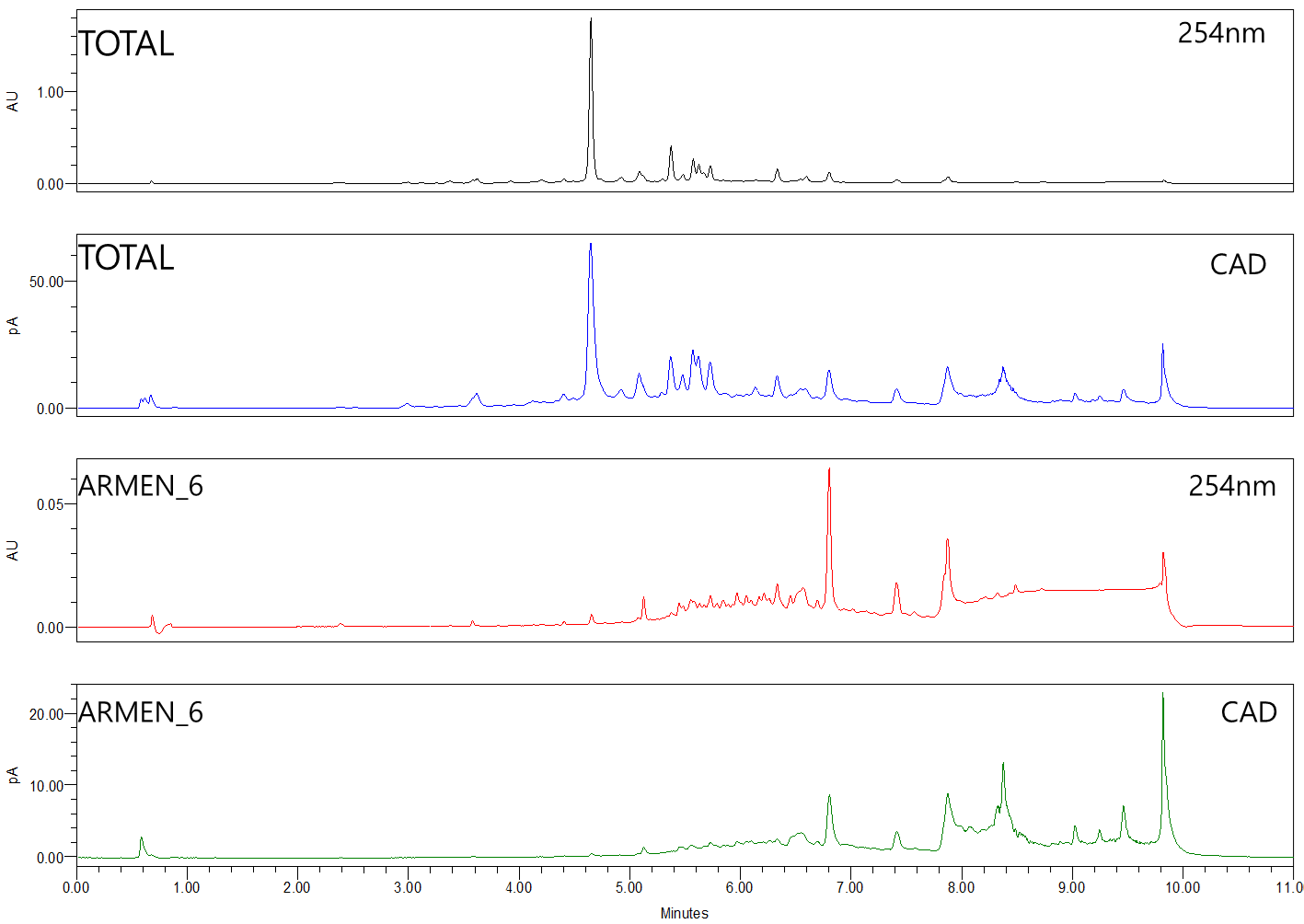


**Figure S2. UPLC-PDA of Frs. 1-6 of *P. rotundum* var. *subintegrum* extract.**

**Figure S3. ^1^H and ^13^C NMR spectrum of Vms.**

**UPLC-QTof-MS analysis**

*P. rotundum* var. *subintegrum* extract profiling was performed using an ACQUITY UPLCTM system (Waters Corporation, Milford, MA, USA) equipped with a binary solvent delivery manager and a sample manager coupled to a Micromass Q-TOF Premier^TM^ mass spectrometer (Waters Corporation) with an electrospray ionisation (ESI) interface with MassLynex V4.1 software. Chromatographic separation was performed using an ACQUITY BEH C18 chromatography column (2.1 × 100 mm, 1.7 μm). The column temperature was maintained at 35°C, and the mobile phases A and B were water with 0.1% formic acid and acetonitrile with 0.1% formic acid, respectively. The gradient elution program was as follows: 0.0-1.0 min, 10% B; 1.0-10.5 min, 10-23% B; 10.5-12.0 min, 23-98% B; wash for 1.4 min with 100% B; and a 1.6 min recycle time. The injection volume was 2.0 μL, and the flow rate was 0.4 mL/min. The mass spectrometer was operated in positive ion mode. N_2_ was used as the desolvation gas. The desolvation temperature was 350°C, the flow rate was 500 L/h, and the source temperature was 110°C. The capillary and cone voltages were 2300 V and 50 V, respectively. The Q-TOF Premier^TM^ was operated in v mode with a 9000 mass resolving power.The data were collected for each test sample from 100 to 1500 Da with 0.25-s scan time and 0.01-s interscan delay over the 15 min analysis time. Leucine-enkephalin was used as the reference compound (*m/z* 554.2661 in the negative mode).

**
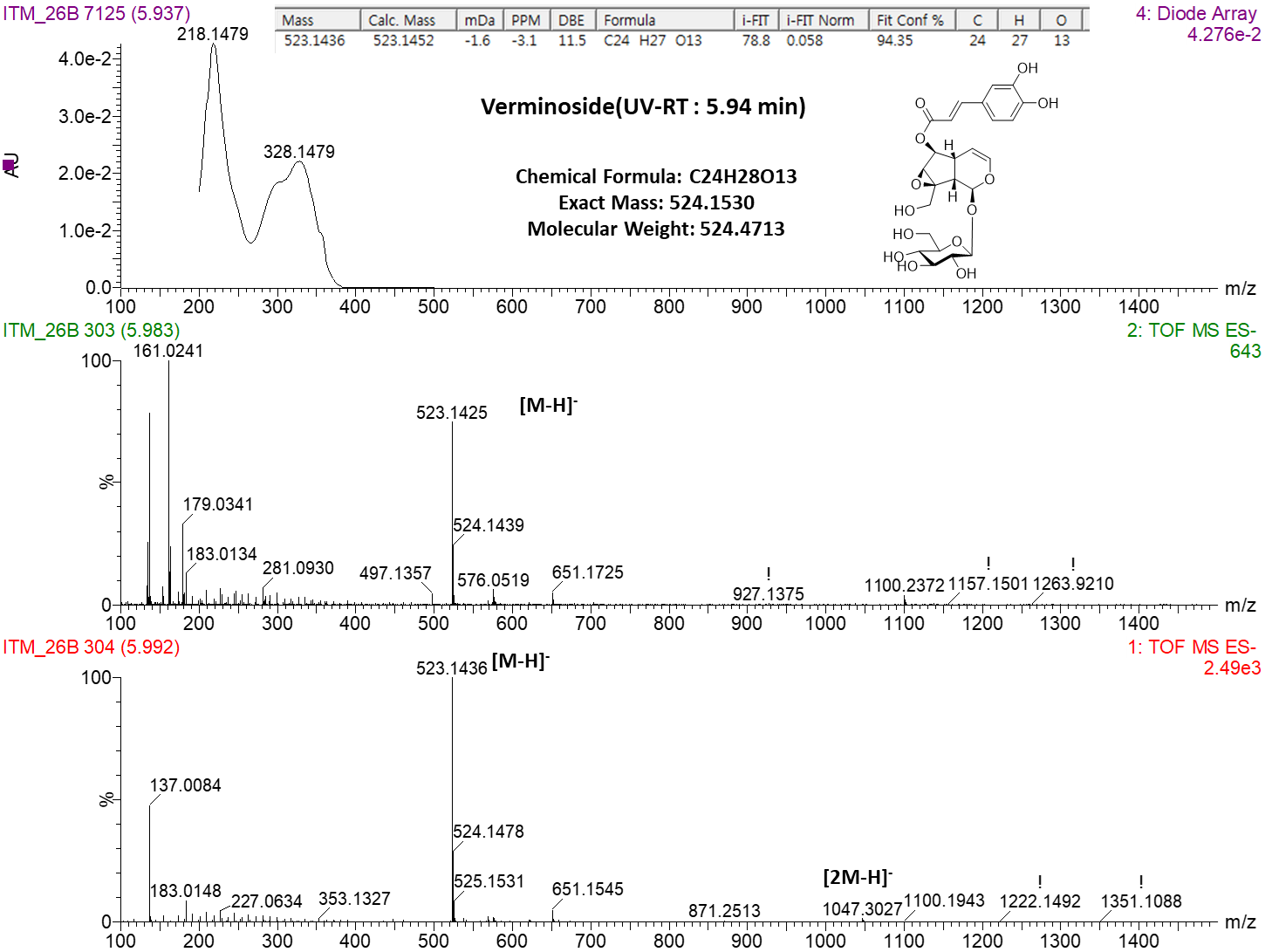
**

**Figure S4. UV, MS/MS, MS and HREIMS data of Vms.**

**
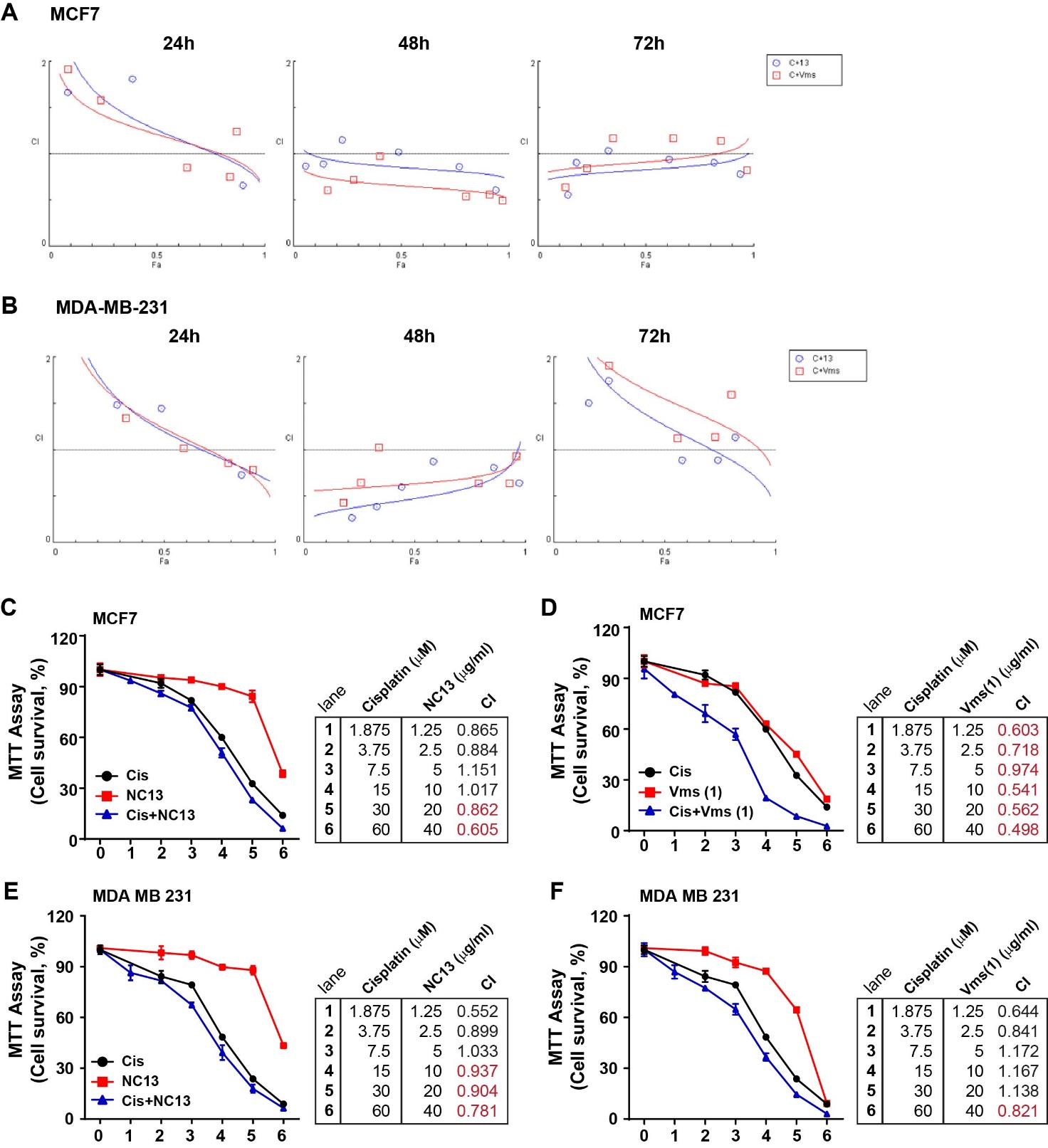
**

**Figure S5. CompuSyn analysis of the NC13 or Vms chemoadjuvant therapy.** Computer-simulated Fa-CI plots of cisplatin and NC13/Vms combination treatment in (A) MCF-7 cells and (B) MDA-MB-231 cells for 24-, 48-, 72-hours. (CI <1, CI =1, CI >1 represent synergism, additive, and antagonism effect, respectively. Fa = Fraction affected, and CI = Combination index) Experimental data points for plotting computer-simulated Fa-CI graph were collected through measuring cellular viability. (C, D) MCF-7 cells and (E, F) MDA-MB-231 cells were treated with 2-fold dilution of cisplatin with NC13 or Vms combination mixture for 48hr. Combination index (CI) was evaluated using Compusyn software.

**
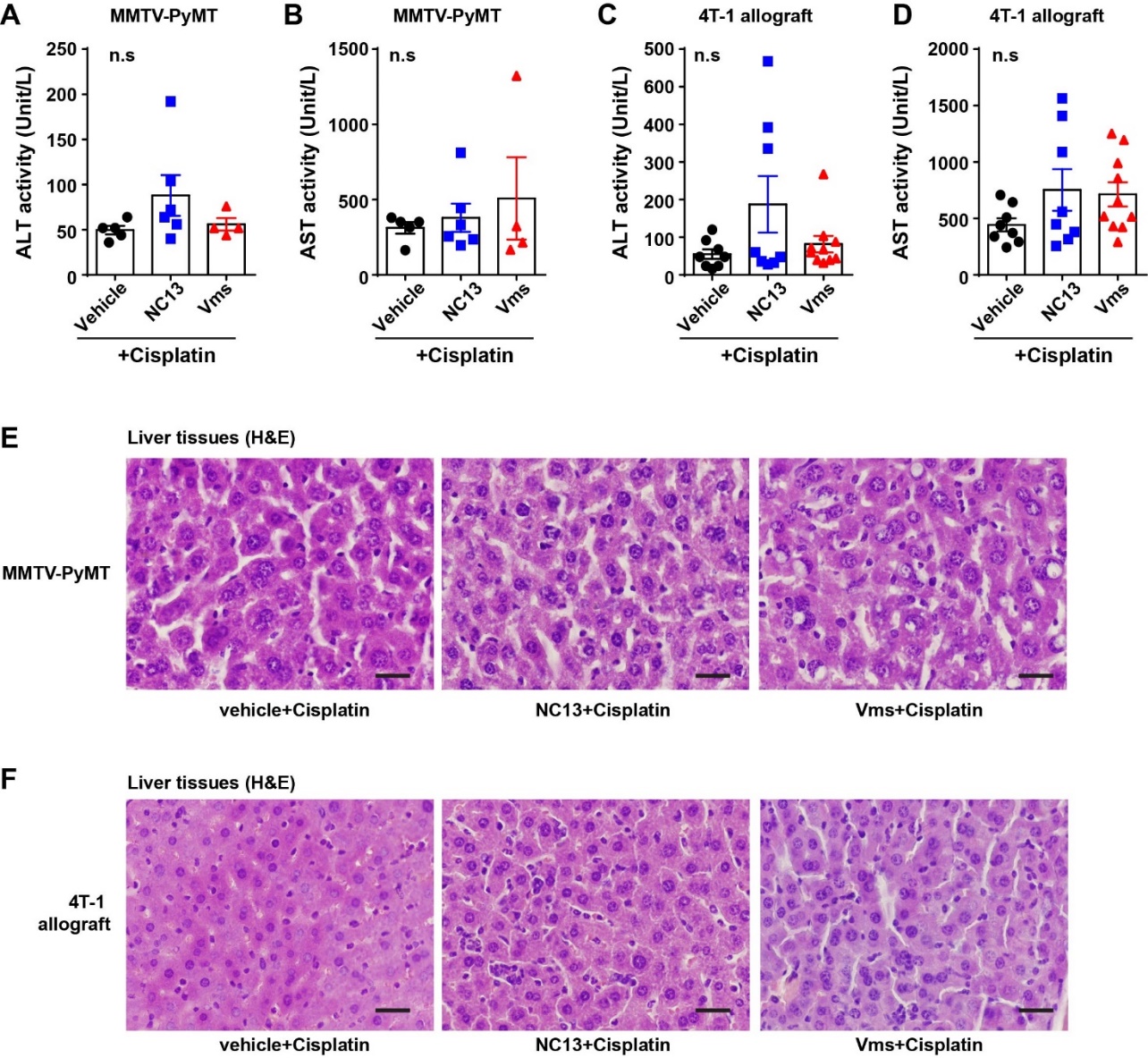
**

**Figure S6. Assessment of drug toxicity in the *in vivo* mouse models**. Serum ALT and AST activity in MMTV-PyMT (A, B) and 4T-1 allograft mice (C, D) in the indicated groups. Representative images of H&E stained sections of liver tissue in MMTV-PyMT (E) and 4T-1 allograft mice (F) in the indicated groups. (Scale bar = 16 μm)

**
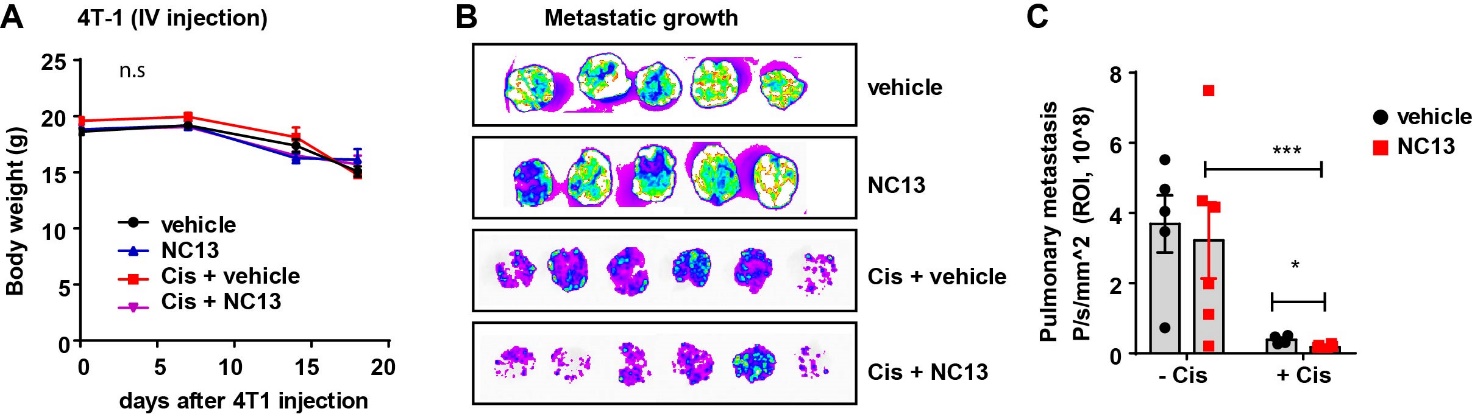
**

**Figure S7. Chemoadjuvant effect of NC13 in cisplatin on the *in vivo* metastasis model.** BALB/C mice were intravenously injected with 4T-1 cells and received indicated treatments up to 18days. (A) Body weight of indicated mice group. (B) Representative fluorescent images of lung metastatic burden are displayed and (C) quantified in each group. Statistical significance was evaluated by one-way ANOVA, followed by *post hoc tukey’s* multiple comparison test. *P < 0.05, **P < 0.01, ***P < 0.001.

**
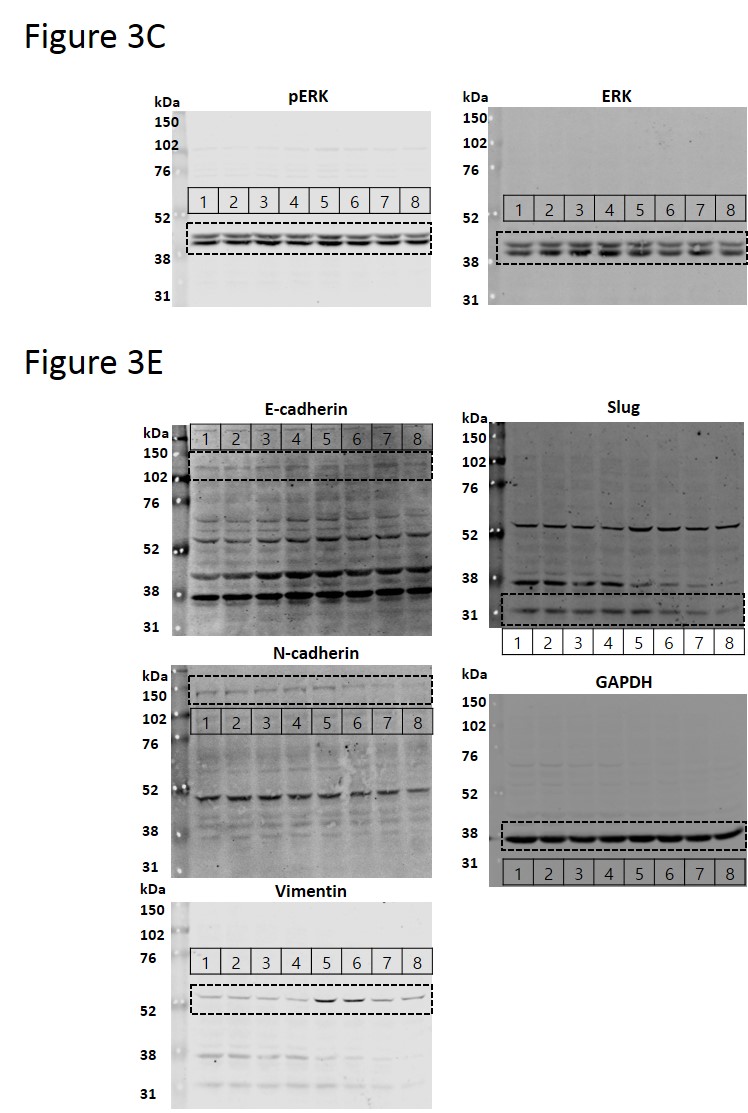
**

**Figure S8.** Uncropped blots corresponding to Figure 3C, E. Identical samples were blotted on different membranes. Squared boxes indicate cropped regions. Lane #1 corresponds to vehicle, lane #2 to NC13, lane #3 to Vms, lane #4 to Rosi, lane #5 to cisplatin, lane #6 to cisplatin and NC13, lane #7 to cisplatin and Vms, and lane #8 to cisplatin and Rosi.

**
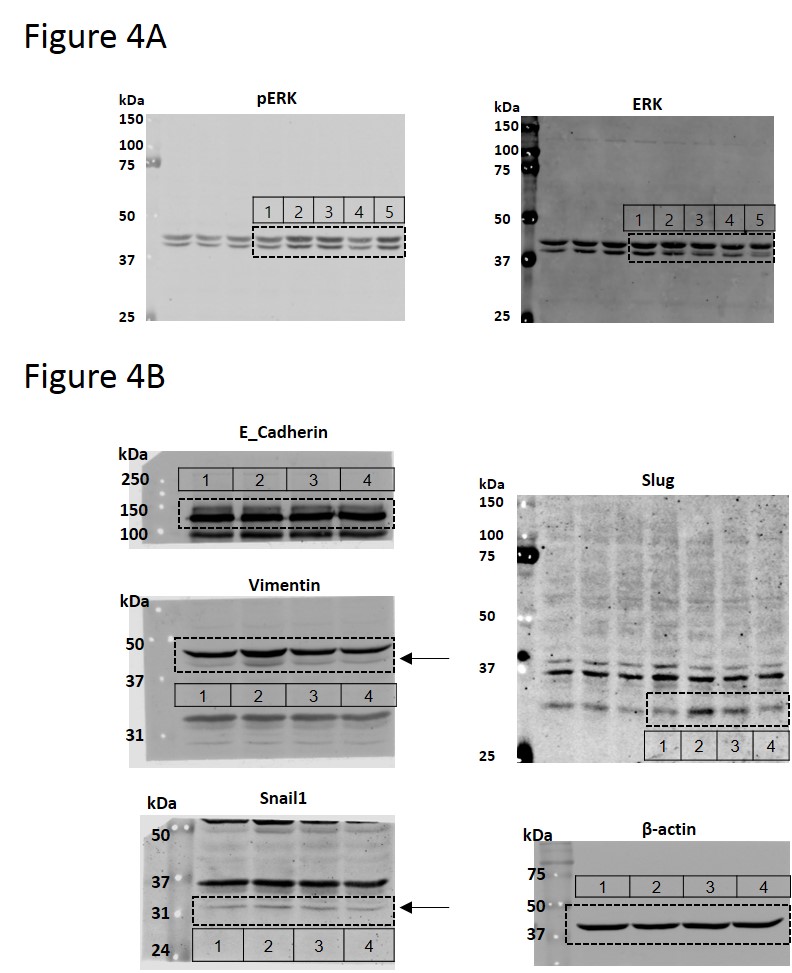
**

**Figure S9.** Uncropped blots corresponding to Figure 4A, B. Identical samples were blotted on different membranes. Squared boxes indicate cropped regions. Lane #1 corresponds to vehicle, lane #2 to cisplatin, lane #3 to cisplatin and NC13, lane #4 to cisplatin and Vms and lane #5 to cisplatin and Rosi. Unlabeled lanes belong to an unrelated experiment.


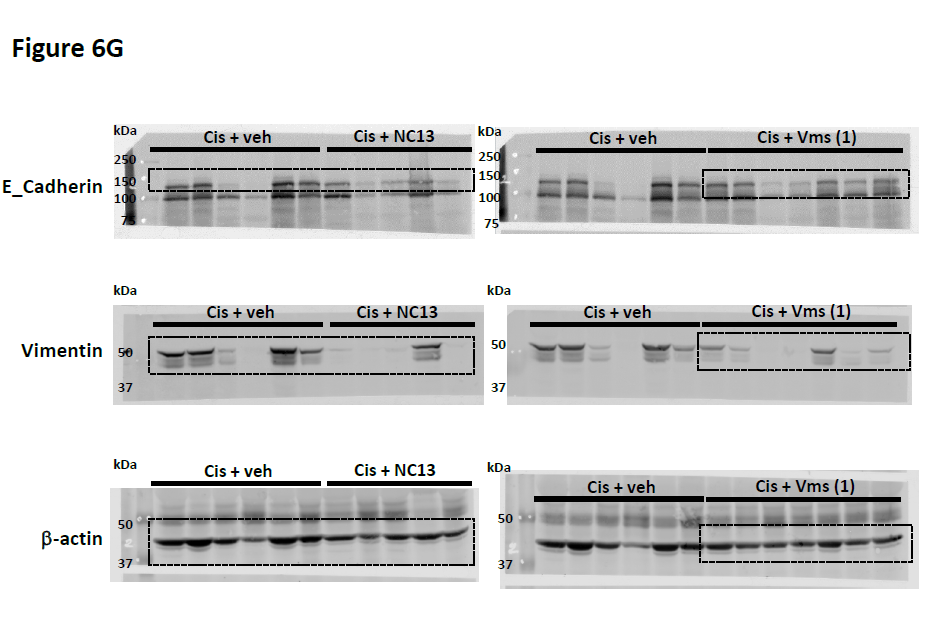


**Figure S10.** Uncropped blots corresponding to Figure 6G. Proteins samples in Cis+Vms group were blotted in a separated membrane with the samples in Cis+NC13 group, while Cis+Veh samples were identical. Squared boxes indicate cropped regions.
